# Four Pentasaccharide Resin Glycosides from *Argyreia acuta*

**DOI:** 10.3390/molecules22030440

**Published:** 2017-03-11

**Authors:** Bang-Wei Yu, Jing-Jing Sun, Jie-Tao Pan, Xiu-Hong Wu, Yong-Qin Yin, You-Shao Yan, Jia-Yan Hu

**Affiliations:** 1 School of Traditional Chinese Medicinal Chemistry, Guangdong Pharmaceutical University, Guangzhou 510006, China; bondbeth@126.com (B.-W.Y.); 13424039203@163.com (J.-J.S.); panjietao@126.com (J.-T.P.); xg661@126.com (Y.-S.Y.); 13424037598@163.com (J.-Y.H.); 2 National TCM Key Lab of Serum Pharmacochemistry, Heilongjiang University of Chinese Medicine, Heping Road 24, Harbin 150040, China

**Keywords:** *Argyreiaacuta*, resin glycosides, structural identification

## Abstract

Four pentasaccharide resin glycosides, acutacoside F–I (**1**–**4**), were isolated from the aerial parts of *Argyreia acuta*. These compounds were characterized as a group of macrolactones of operculinic acid A, and their lactonization site of 11*S*-hydroxyhexadecanoic acid was esterified at the second saccharide moiety (Rhamnose) at C-2. The absolute configuration of the aglycone was *S*. Their structures were elucidated by established spectroscopic and chemical methods.

## 1. Introduction

Resin glycosides, found mostly in plants of the morning glory family (Convolvulaceae), whose structures include fatty acid aglycone and oligosaccharide groups, were partially esterified with different fatty acids. Hundreds of resin glycosides have been isolated from different genera, including *Ipomoea* [1], *Merremia* [2], and *Pharbitis* [3], some of which have potential pharmacological activities, such as phytogrowth-inhibition [4], antifungal [5], cytotoxicity [6], and antibacterial [7], effects on the central nervous system [8], as well as multi-drug efflux pumps blocking effects [7,9,10,11].

*Argyreia acuta* Lour. (Convolvlaceae) is a climbing shrub, known as twining vine. It is widely distributed in Guangdong and Guangxi province in China, and is used as folk medicine for dispelling wind; eliminating dampness; relieving cough; reducing sputum; stopping bleeding and promoting tissue regeneration; relaxing and activating the tendons; and removing toxicity to eliminate carbuncles. Saponins, steroids, fatty acids [12], flavonoids, courmains, cardiac glycosides and phenolics [13], have been found in this species. Our previous studies reported that five resin glycosides, acutacoside A–E, were obtained from the plant, and some of which showed potential inhibition against α-glucosidase. [14,15]. As a part of our ongoing chemical studies on the resin glycosides from Convolvlaceae, four new partially acylated pentasaccharide resin glycosides, designated as acutacoside F–H, were isolated from *A. acuta*. These new compounds, macrolactones of operculinic acid A, were partially esterified with different fatty acids. The lactonization site of the agylcone, jalpinolic acid, was attached to the second saccharide moiety (Rhamnose) at C-2. Their structures were elucidated on the basis of extensive spectroscopic data interpretation and chemical degradation.

## 2. Results and Discussion

Compounds **1**–**4** were separated from the dried aerial parts of *A. acuta* with several chromatograph methods.

**Figure 1:**
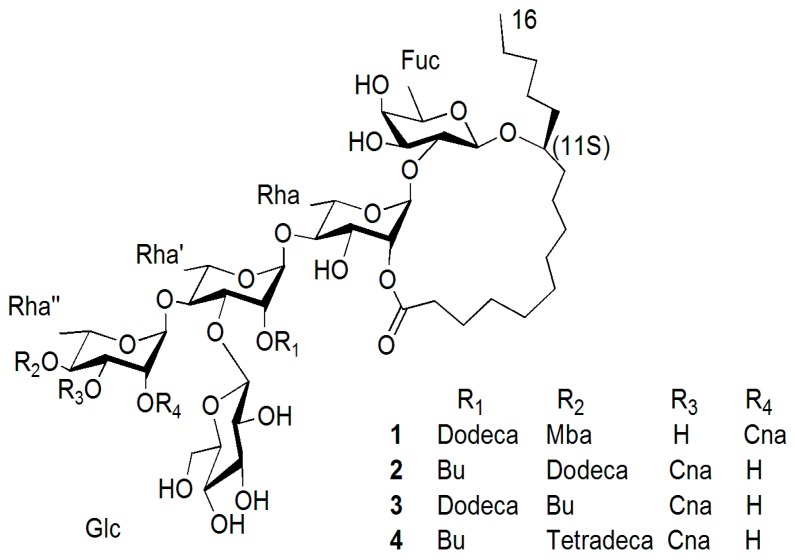
Structures of compounds **1**–**4**.

Acutacoside F (**1**), obtained as a white amorphous powder, was found to have the molecular formula C_72_H_116_O_26_ on the basis of HRTOFMS (positive mode [M + Na]^+^ peak at *m/z* 1419.7766, calcd. for C_72_H_116_O_26_Na, 1419.7653). The UV spectrum of compound **1** revealed an absorption band at 278 (0.67) nm. Its IR spectrum displayed absorptions of hydroxyl (3429 cm^−1^), alkyl (2929 cm^−1^, 2859 cm^−1^), carbonyl (1730 cm^−1^), and aromatic (1684 cm^−1^). Compound **1** was alkaline hydrolyzed and detected after methylation by GC-MS; three organic acids and operculinic acid A (**5**) [14] were afforded. Subsequent acidic hydrolysis of the glycosidic acid liberated fucose, glucose and rhamnose, which were identified as d-fucose, l-rhamnose and d-glucose by comparison with those of authentic samples by the GC-MS method. The organic layer obtained from alkaline hydrolysis of **1** was methylated and identified by GC-MS analysis. The 2-Methylbutyric acid methyl ester (*t*_R_ 4.39 min) *m/z* [M + H]^+^ 117 (5), 101 (23), 88 (96), 57 (100), 41 (55), 29 (45), 27 (19); *trans*-cinnamic acid methyl ester (*t*_R_ 13.29 min) *m/z* [M]^+^ 162 (40), 131 (100), 103 (66), 77 (32); and *n*-dodecanoic acid methyl ester (*t*_R_ 15.17 min) *m/z* [M]^+^ 200 (1), 172 (1), 168 (10), 157 (15), 143(18), 129 (7), 87 (64), 74 (100), 55 (25), 43 (20), 41 (18) were identified. The 2-methylbutyric acid was proven to have an *S* configuration [16]. The ^1^H-NMR spectrum of **1** revealed the presence of one benzene ring δ_H_ 7.30–7.36 (5H, m); one *trans*-olefinic bond δ_H_ 6.66 (1H, d, *J* = 16.0) and 7.83 (1H, d, *J* = 16.0); and five sugar units in the molecule [17,18]. The ^13^C-NMR spectrum of compound **1** showed five anomeric carbon signals at δ_C_ 104.3, 98.5, 99.2, 100.2 and 105.2. These chemical shifts were different from acutacoside A and acutacoside B but the same as the core of operculinic acid A; according to the data summary [19] of the resin glycosides, one of the three organic acid groups was esterificated at Rha’-C-2. Five anomeric hydrogen chemical shifts were obtained at δ_H_ 4.78 (1H, d, *J* = 7.0 Hz), 5.63 (1H, br s), 5.80 (1H, br s), 6.58 (1H, br s) and 5.01 (1H, d, *J* = 7.8 Hz) by the HSQC spectrum. Then each monosaccharide unit was established by TOCSY experiments, and the correlative carbons [20] were assigned by HSQC spectrum data. The correlation sites of monosaccharides in the glycosidic acid were known [14], which were between H-1 of Rha and C-2 of Fuc; H-1 of Rha’ and C-4 of Rha; H-1 of Rha” and C-4 of Rha’; H-1 of Glc’ and C-3 of Rha’; and H-1 of Fuc and C-11 of the 11-hydroxyhexadecanoyl moiety (aglycone), see Figure S1. The organic acid groups and lactonization sits were also assigned by HMBC spectrum data. The organic acid groups’ correlations were at H-2 of Rha” to C-1 of can; H-4 of Rha” to C-1 of Mba; and H-2 of Rha’ to C-1 of Dodeca; and the lactonization site was at C-2 of Rha. Analysis of the TOCSY, HSQC, and HMBC spectrum of compound **1** allowed for the complete assignment of the ^1^H- and ^13^C-NMR spectral data (Table 1). Consequently, the structure of compound **1** was determined to be (*S*)-jalapinolic acid 11-*O*-β-d-glucopyranosyl-(1→3)-*O*-[2-O-trans-cinnamoyl-4-O-(S)-2-methylbutyryl-α-l-rhamnopyranosyl-(1→4)]-*O*-[2-O-n-dodecanoyl]-α-l-rhamnopyranosyl-(1→4)-*O*-α-l-rhamnopyranosyl-(1→2)-*O*-β-d-fucopyranoside, intramolecular 1,2′′-ester (Figure 1).

Acutacoside G–I (**2**–**4**) afforded white, amorphous powders, and gave quasi-molecular ions at *m/z* 1405.7697 [M + Na]^+^, 1405.7466 [M + Na]^+^ and 1433.8016 [M + Na]^+^ in HRTOFMS, which suggested the molecular formulas C_71_H_114_O_26_ (calcd for C_71_H_114_O_26_Na: 1405.7496), C_71_H_114_O_26_ (calcd. for C_71_H_114_O_26_Na: 1405.7496) and C_73_H_118_O_26_ (calcd. for C_73_H_118_O_26_Na:1433.7809). The IR spectrum gave absorption bands of hydroxyl groups at 3451, 3424 and, 3453 cm^−1^ and carbonyl groups at 1733, 1728 and 1734 cm^−1^. Analysis of the TCOSY, HSQC, and HMBC spectra of compounds **2**–**4** allowed for the complete assignment of the ^1^H- and ^13^C-NMR spectral data (Table 1). Independent alkaline hydrolysis of **2**–**4** afforded a mixture of organic acids and a glycosidic acid, respectively. A butyric acid group and a *trans*-olefinic acid group were found in **2**–**4**; a *n*-dodecanoic acid was found in **2** and **3**; and a *n*-tetradecanoic acid methyl ester (*t*_R_ 18.81 min) was found in **4** by GC-MS experiments. The glycosidic acid is operculinic acid A, which was obtained from alkaline hydrolysis of **2**–**4**. The key HMBC correlations confirmed the esterification positions of the acyl residues in the oligosaccharide core, thus a *trans*-cinnamoyl group was located at C-3 of Rha” in **2**–**4**; a butyl group was located at C-2 of Rha’ in **2** and **4**, and located at C-4 of Rha” in **3**; a *n*-dodecanoyl group was located at C-4 of Rha” in **2**; and a *n*-tetradecanoyl group was located at C-4 of Rha" in **4**. The lactonization position of the aglycone was C-2 of Rha for **2**–**4**. The structure of compound **2** was determined to be (*S*)-jalapinolic acid 11-*O*-β-d-glucopyranosyl-(1→3)-*O*-[3-O-trans-cinnamoyl-4-O-(S)-n-dodecanoyl-α-l-rhamnopyranosyl-(1→4)]-O-[2-O-butyryl]-α-l-rhamnopyranosyl-(1→4)-*O*-α-l-rhamnopyranosyl-(1→2)-*O*-β-d-fu-copyranoside, intramolecular 1,2”-ester; and the structure of compound **3** was suggested as (*S*)-jalapinolic acid 11-*O*-β-d-glucopyranosyl-(1→3)-*O*-[3-O-trans-cinn-amoyl-4-O-butyryl-α-l-rhamnopyranosyl-(1→4)]-O-[2-O-n-dodecanoyl]-α-l-rhamn-opyranos-yl-(1→4)-*O*-α-l-rhamnopyranosyl-(1→2)-*O*-β-d-fucopyranoside, intramolecular 1,2”-ester; and the structure of compound **4** was suggested as (*S*)-jalapinolic acid 11-*O*-β-d-glucopyranosyl-(1→3)-*O*-[3-O-trans-cinnamoyl-4-O-n-tetradecanoyl-α-l-rhamnopyranosyl-(1→4)]-O-[2-O-butyryl]-α-l-rhamnopyranosyl-(1→4)-*O*-α-l-rhamnopyranosyl-(1→2)-*O-*β-d-fucopyranoside, intramolecular 1,2”-ester (Figure 1).

## 3. Experimental Section

### 3.1. General

IR spectra were taken from KBr disks on a Shimadzu FTIR spectrophotometer (Shimadzu Corp., Kyoto, Japan). The UV spectrum were recorded on a Shimadzu UV-2550 spectrophotometer (Shimadzu Corp., Kyoto, Japan). All of the ^1^H- and ^13^C-NMR spectra were recorded on an INOVA 500 spectrometer (Varian, Palo Alto, CA, USA), using tetramethylsilane (TMS) as an internal standard. Two-dimensional NMR spectra include total correlation spectroscopy (TOCSY), heteronuclear single quantum coherence (HSQC), and heteronuclear multiple-bond coherence (HMBC). The chemical shifts in the NMR spectrum were recorded as δ values. HR-TOF-MS experiments were performed on an AB SCIEX Triple TOF 5600 plus MS spectrometer (Applied Biosystems, Foster, CA, USA). Preparative high-performance liquid chromatography (PHPLC) was performed using a Shimadzu LC-6AD series instrument (Shimadzu Corp., Kyoto, Japan) equipped with a UV detector at 280 nm and a Shim-Park RP-C_18_ column (20 × 200 mm i.d.). GC-MS experiments were performed on a TRACE GC ULTRA DSQ II instrument (Thermo Electron, Beverly, MA, USA). Optical rotations were measured with an Anton Paar-MCP600 polarimeter in MeOH solution. The centrifugation was applied with D05 (Hunan Hexi Instrument Co., Ltd., Changsha, China). Adsorbents for column chromatography were silica gel (200–300 µm, Qingdao Marine Chemical Co., Ltd., Qingdao, China), Sephadex LH-20 (75–150 µm, Pharmacia, Uppsala, Sweden), all of the chemicals and solvents used in the current study were of analytical grade.

### 3.2. Plant Material

The aerial parts of *Argyreia acuta* were collected in April 2014 from the Yulin city of the Guangxi Province, China. The plant material was identified by Associate Professor H.-Y. Ma in Guangdong Pharmaceutical University (Guangzhou, China). A voucher specimen (No. 201404) was deposited at School of Traditional Chinese Medicinal Chemistry, Guangdong Pharmaceutical University.

### 3.3. Extraction and Isolation

Dried aerial parts (30 kg) of *A*. *acuta* were cut to small pieces and were extracted two times with 95% EtOH under reflux for 2 h and concentrated under vacuum, which was then extracted three times sequentially with equal volumes of petroleum ether and chloroform extract (150 g), which was separated into five fractions (A–E) by normal-phase silica gel column chromatography (CC) (1200 g of silica gel, 200–300 mesh) using a stepwise gradient elution of CHCl_3_/MeOH (from 100:0 to 0:100, *v/v*). Fracton B (7.3 g) was separated into three subfractions (B-1, B-2 and B-3) on a normal-phase silica gel column using a stepwise gradient elution of petroleum ether/acetone (from 100:0 to 50:50, *v/v*). Fraction B-1 was then passed through a Sephadex LH-20 column with a MeOH eluent to yield three subfractions (B-1-1, B-1-2 and B-1-3); the B-1-1 subfraction was purified by a reverse-phase HPLC system (10 mL/min, 280 nm), eluted with MeOH/H_2_O (99:1, *v/v*) to afford **1** (9.5 mg, *t*_R_ 35.37 min); **2** (8 mg, *t*_R_ 42.61 min); **3** (6 mg, *t*_R_ 32.05 min) and **4** (10 mg, *t*_R_ 43.75 min).

### 3.4. Spectral Data

*Acutacoside F* (**1**)*:* White amorphous powder, ${\left[\alpha \right]}_{D}^{25}$ −24.4° (*c* 0.09, MeOH); UV (MeOH) λ_max_ (log ε) 278 (0.67) nm; IR (KBr) ν_max_: 3429, 2929, 2859, 1730, 1684, 1141 and 1061 cm^−1^, ^1^H-NMR and ^13^C-NMR data, see Table 1; HR-TOF-MS *m/z* 1419.7766 [M + Na]^+^ (calcd. for C_72_H_116_O_26_Na, 1419.7653).
*Acutacoside G* (**2**): White amorphous powder; ${\left[\alpha \right]}_{D}^{25}$ −18.0° (*c* 0.25, MeOH); UV (MeOH) λ_max_ (log ε) 217 (0.74), 279 (1.02) nm; IR (KBr) ν_max_: 3451, 2930, 1733 and 1064 cm^−1^, ^1^H-NMR and ^13^C-NMR data, see Table 1; HR-TOF-MS *m/z* 1405.7697 [M + Na]^+^ (calcd. for C_71_H_114_O_26_Na, 1405.7496).
*Acutacoside H* (**3**): White amorphous powder; ${\left[\alpha \right]}_{D}^{25}$ −23.7° (*c* 0.19, MeOH); UV (MeOH) λ_max_ (log ε) 217 (0.37), 280 (0.42) nm; IR (KBr) ν_max_: 3424, 2929, 1728 and 1067 cm^−1^, ^1^H-NMR and ^13^C-NMR data, see Table 1; HR-TOF-MS *m/z* 1405.7466 [M + Na]^+^ (calcd. for C_71_H_114_O_26_Na, 1405.7496).
*Acutacoside I* (**4**): White amorphous powder; ${\left[\alpha \right]}_{D}^{25}$ −11.3° (*c* 0.15, MeOH); UV (MeOH) λ_max_ (log ε) 217 (0.62), 280 (0.87) nm; IR (KBr) ν_max_: 3453, 2930, 1734 and 1066 cm^−1^, ^1^H-NMR and ^13^C-NMR data, see Table 1; HR-TOF-MS *m/z* 1433.8016 [M + Na]^+^ (calcd. for C_73_H_118_O_26_Na, 1433.7809).

### 3.5. Hydrolysis

In order to identify the kinds of organic acid groups, sugar and the absolute configuration of aglycone, compounds **1**–**4** were hydrolyzed with alkaline and acid. The procedures were performed as described earlier [12].

## 4. Conclusions

In conclusion, investigation of the aerial parts of *A. acuta* afforded four new compounds.

## Figures and Tables

**Table 1:** NMR data for compounds **1**–**4** in pyridine-*d*_5_.

Position	1		2		3		4	
	^13^C	^1^H	^13^C	^1^H	^13^C	^1^H	^13^C	^1^H
Fuc-1	104.6	4.78 d (7.0)	104.4	4.73 d (7.5)	104.6	4.72 d (7.2)	104.0	4.72 d (7.5)
2	80.2	4.19 dd (7.0, 9.5)	79.7	4.15 dd (7.5, 9.5)	80.2	4.17 dd (7.2, 9.4)	79.7	4.16 dd (7.5, 9.5)
3	73.6	4.15 dd (9.5, 3.0)	73.2	4.03 *	73.7	4.14 dd (9.4, 3.0)	72.8	4.04 *
4	73.0	3.98 d (3.0)	72.1	3.90 *	73.2	3.96 d (3.0)	72.7	3.90 *
5	70.8	3.77 br q (6.5)	71.1	3.73 br q (6.5)	71.1	3.74 br q (6.6)	70.6	3.73 br q (6.5)
6	17.4	1.52 d (6.0)	16.7	1.48 d (6.5)	17.7	1.50 d (6.0)	16.7	1.49 d (6.5)
Rha-1	98.6	5.53 br s	98.3	5.50 br s	98.8	5.51 br s	98.3	5.52 br s
2	73.4	5.95 br s	73.2	5.92 br s	73.7	5.93 br s	73.2	5.93 br s
3	73.2	5.03 dd (3.0, 9.0)	68.7	5.02 dd (3.0, 9.0)	69.3	5.03 dd (3.3, 9.3)	68.7	5.01 dd (3.0, 9.0)
4	82.0	4.19 *	82.0	4.16 dd (9.0, 9.0)	82.5	4.18 *	82.1	4.16 dd (9.0, 9.0)
5	69.2	4.48 *	68.3	4.47 dd (9.0, 5.0)	68.5	4.37 *	68.3	4.47 dd (9.0, 5.0)
6	19.0	1.58 d (5.4)	18.9	1.63 d (5.0)	19.5	1.63 d (5.4)	18.9	1.63 d (5.0)
Rha′-1	99.3	5.80 br s	100.1	5.82 br s	100.6	5.84 br s	100.1	5.82 br s
2	73.2	6.32 br s	73.4	6.31 br s	73.9	6.33 br s	73.4	6.30 br s
3	79.1	4.79 *	78.8	4.78 *	79.3	4.79 dd (2.9, 9.2)	78.7	4.78 *
4	79.9	4.36 *	79.6	4.35 *	80.1	4.36 dd (9.2, 9.2)	79.7	4.35 *
5	69.0	4.52 *	68.0	4.50 *	68.4	4.50 dd (9.2, 6.5)	67.7	4.50 *
6	19.1	1.63 d (6.0)	19.1	1.64 d (6.5)	19.4	1.65 d (6.0)	18.8	1.64 d (6.5)
Rha″-1	100.3	6.58 br s	103.2	6.27 br s	103.7	6.27 br s	103.2	6.26 br s
2	70.8	6.37 br s	69.1	5.25 br s	69.5	5.26 br s	69.1	5.26 br s
3	68.2	6.00 dd (3.1, 10.0)	71.5	6.00 dd (3.0, 10.0)	72.0	6.01 dd (3.1, 10.0)	71.5	6.00 dd (3.0, 10.0)
4	73.0	4.09 *	71.3	6.08 dd (10.0, 10.0)	71.8	6.09 dd (10.0, 10.0)	71.3	6.08 dd (10.0, 10.0)
5	68.4	4.37 *	69.7	4.44 *	70.2	4.48 dd (10.0, 6.2)	69.7	4.47 *
6	18.4	1.77 d (6.3)	17.7	1.42 d (6.5)	18.2	1.43 d (6.2)	17.7	1.42 d (6.5)
Glc-1	105.6	5.01 d (7.8)	105.0	5.07 d (7.5)	105.8	5.09 d (7.8)	105.3	5.08 d (7.5)
2	75.0	3.90 dd (7.8, 9.0)	74.9	3.97 *	75.5	3.95 dd (7.8, 9.0)	74.9	3.97 *
3	78.3	4.07 *	78.2	4.10 *	78.7	4.08 dd *	78.2	4.10 *
4	71.5	3.92 *	68.3	3.93 *	68.7	3.94 *	68.0	3.93 *
5	78.2	3.85 *	77.9	3.83 m	78.4	3.81 *	77.5	3.85 m
6	63.2	4.05 *	62.5	4.09 *	63.2	4.09 *	62.5	4.09 *
		4.32 *		4.40 *		4.43 *		4.40 *
Ag-1	173.5		173.3		173.4		173.3	
2	34.7	2.29 m	34.3	2.27 m	33.5	2.23 m	34.3	2.29 m
		2.46 m		2.44 m		2.40 m		2.45 m
11	82.4	3.86 m	82.2	3.80 m	82.7	3.83 m	82.2	3.82 m
16	14.7	0.86 *	14.1	0.83 t (7.0)	14.6	0.86 *	14.1	0.84 t (7.0)
Cna-1	166.5		166.3		166.8		166.3	
2	118.9	6.66 d (16.0)	118.5	6.58 d (16.0)	118.9	6.66 d (16.0)	118.3	6.58 d (16.0)
3	146.7	7.83 d (16.0)	145.2	7.85 d (16.0)	145.7	7.86 d (16.0)	145.3	7.85 d (16.0)
1′	134.7		135.3		135.0		135.3	
2′ and 6′	128.5	7.36 m	128.6	7.43 m	128.8	7.42 m	128.4	7.43 m
3′ and 5′	129.3	7.30 m	128.9	7.32 m	129.6	7.33 m	129.1	7.33 m
4′	130.3	7.30 m	130.8	7.32 m	131.1	7.33 m	131.0	7.33 m
Dodeca-1	174.0		173.4		173.4			
2	34.4	2.32 *	34.2	2.48 m	34.9	2.34 *		
12	14.7	0.87 *	14.1	0.83 t (7.0)	14.6	0.86 *		
Mba-1	176.6							
2	41.7	2.46 m						
2-CH_3_	16.7	1.23 d (7.0)						
4	12.1	0.86 t (7.0)						
Bu-1			175.8	7.32 m	174.8		175.8	
2			34.0	2.30 m	34.8	2.38 t (7.8)	34.0	2.26 m
4			14.1	0.83 t (7.0)	14.6	0.86 *	14.1	0.84 t (7.0)
Tetradeca-1							173.4	
2							34.2	2.53
14							14.1	0.84 t (7.0)

Chemical shifts (δ) are in ppm relative to TMS. The spin coupling (*J*) is given in parentheses (Hz). Chemical shifts marked with an asterisk (*) indicate overlapped signals. Spin-coupled patterns are designated as follows: br s = broad singlet, d = doublet, t = triplet, m = multiplet, q = quartet. Abbreviations: Glc = glucose; Rha = rhamnose; Ag = 11-hydroxyhexadecanoyl; Mba = 2*S*-methylbutanoyl; Cna = *trans*-cinnamoyl; Bu = butyryl; Dodeca = *n*-dodecanoyl; Tetradeca = *n*-tetradecanoyl.

## Supplementary Materials

The following are available online, alongside Figures S1–S17.
