# Supplementary material for: Four Pentasaccharide Resin Glycosides from Argyreia acuta
Source: Molecules. 2017 Mar 11;22(3):440. doi: 10.3390/molecules22030440 (PMC6155385; doi:10.3390/molecules22030440)
Supplement: Supplementary file 1 [file molecules-22-00440-s001.pdf]

SUPPLEMENTARY MATERIAL

Four pentasaccharide resin glycosides from *Argyreia acuta*

Bang-wei Yu[1], Jing-Jing Sun[1], Jie-tao Pan[1], Xiu-Hong Wu[2],*, Yong-Qin Yin[1],*, You-shaoYan[1], Jia-Yan Hu[1]

[1] School of Traditional Chinese Medicinal Chemistry, Guangdong Pharmaceutical University, Guangzhou 510006, People's Republic China; bondbeth@126.com (B.-W.Y.); 13424039203@163.com (J-J.S.); panjietao@126.com (J-T.P.); yys-003@hotmail.com (Y.-S.Y.); 13424037598@163.com (J.-Y.H.)

[2] National TCM Key Lab of Serum Pharmacochemistry, Heilongjiang University of Chinese Medicin, Heping Road 24, Harbin 150040, China

* Correspondence: wxh8088@163.com; yongqinyin@126.com;

Tel.: +86-20-39352179; Fax: +86-20-39352174.

Four pentasaccharide resin glycosides from *Argyreia acuta*

Abstract

Four pentasaccharide resin glycosides, acutacoside F-I (**1**–**4**), were isolated from the aerial parts of *Argyreia acuta*. These compounds were characterized as a group of macrolactones of operculinic acid A, and their lactonization site of 11*S*- hydroxyhexadecanoic acid was esterfied the second saccharide moiety (Rhamnose) at C-2, The absolute configuration of the aglycone was *S*. Their structures were established by spectroscopic and chemical methods.

Keywords: *Argyreia acuta*; resin glycosides; structural identification

Table 1. NMR Data for Compounds **1-4** in pyridine-d_5.

Position	1		2		3		4	
	^{13}C	^{1}H	^{13}C	^{1}H	^{13}C	^{1}H	^{13}C	^{1}H
Fuc-1	104.6	4.78 d (7.0)	104.4	4.73 d (7.5)	104.6	4.72 d (7.2)	104.0	4.72 d (7.5)
2	80.2	4.19 dd (7.0, 9.5)	79.7	4.15 dd (7.5, 9.5)	80.2	4.17 dd (7.2, 9.4)	79.7	4.16 dd (7.5, 9.5)
3	73.6	4.15 dd (9.5, 3.0)	73.2	4.03 *	73.7	4.14 dd (9.4, 3.0)	72.8	4.04 *
4	73.0	3.98 d (3.0）	72.1	3.90 *	73.2	3.96 d (3.0）	72.7	3.90 *
5	70.8	3.77 br q (6.5)	71.1	3.73 br q (6.5)	71.1	3.74 br q (6.6)	70.6	3.73 br q (6.5)
6	17.4	1.52 d (6.0)	16.7	1.48 d (6.5)	17.7	1.50 d (6.0)	16.7	1.49 d (6.5)
Rha-1	98.6	5.53 br s	98.3	5.50 br s	98.8	5.51 br s	98.3	5.52 br s
2	73.4	5.95 br s	73.2	5.92 br s	73.7	5.93 br s	73.2	5.93 br s
3	73.2	5.03 dd (3.0, 9.0)	68.7	5.02 dd (3.0, 9.0)	69.3	5.03 dd (3.3, 9.3)	68.7	5.01 dd (3.0, 9.0)
4	82.0	4.19*	82.0	4.16 dd (9.0, 9.0)	82.5	4.18*	82.1	4.16 dd (9.0, 9.0)
5	69.2	4.48 *	68.3	4.47 dd (9.0, 5.0)	68.5	4.37 *	68.3	4.47 dd (9.0, 5.0)
6	19.0	1.58 d (5.4)	18.9	1.63 d (5.0)	19.5	1.63 d (5.4)	18.9	1.63 d (5.0)
Rha'-1	99.3	5.80 br s	100.1	5.82 br s	100.6	5.84 br s	100.1	5.82 br s
2	73.2	6.32 br s	73.4	6.31 br s	73.9	6.33 br s	73.4	6.30 br s
3	79.1	4.79 *	78.8	4.78 *	79.3	4.79 dd (2.9, 9.2)	78.7	4.78 *
4	79.9	4.36 *	79.6	4.35 *	80.1	4.36 dd (9.2, 9.2)	79.7	4.35 *
5	69.0	4.52 *	68.0	4.50 *	68.4	4.50 dd (9.2, 6.5)	67.7	4.50 *
6	19.1	1.63 d (6.0)	19.1	1.64 d (6.5)	19.4	1.65 d (6.0)	18.8	1.64 d (6.5)
Rha''-1	100.3	6.58 br s	103.2	6.27 br s	103.7	6.27 br s	103.2	6.26 br s
2	70.8	6.37 br s	69.1	5.25 br s	69.5	5.26 br s	69.1	5.26 br s
3	68.2	6.00 dd (3.1, 10.0)	71.5	6.00 dd (3.0, 10.0)	72.0	6.01 dd (3.1, 10.0)	71.5	6.00 dd (3.0, 10.0)
4	73.0	4.09 *	71.3	6.08 dd (10.0, 10.0)	71.8	6.09 dd (10.0, 10.0)	71.3	6.08 dd (10.0, 10.0)
5	68.4	4.37 *	69.7	4.44 *	70.2	4.48 dd (10.0, 6.2)	69.7	4.47 *
6	18.4	1.77 d (6.3)	17.7	1.42 d (6.5)	18.2	1.43 d (6.2)	17.7	1.42 d (6.5)
Glc'-1	105.6	5.01 d (7.8)	105.0	5.07 d (7.5)	105.8	5.09 d (7.8)	105.3	5.08 d (7.5)
2	75.0	3.90 dd (7.8, 9.0)	74.9	3.97 *	75.5	3.95 dd (7.8, 9.0)	74.9	3.97 *
3	78.3	4.07 *	78.2	4.10 *	78.7	4.08 dd*	78.2	4.10 *
4	71.5	3.92 *	68.3	3.93 *	68.7	3.94 *	68.0	3.93*
5	78.2	3.85 *	77.9	3.83 m	78.4	3.81 *	77.5	3.85 m
6	63.2	4.05 *	62.5	4.09 *	63.2	4.09 *	62.5	4.09 *
		4.32 *		4.40 *		4.43 *		4.40 *
Ag-1	173.5		173.3		173.4		173.3	
2	34.7	2.29 m	34.3	2.27 m	33.5	2.23 m	34.3	2.29 m
		2.46 m		2.44 m		2.40 m		2.45 m
11	82.4	3.86 m	82.2	3.80 m	82.7	3.83 m	82.2	3.82 m
16	14.7	0.86 *	14.1	0.83 t (7.0)	14.6	0.86 *	14.1	0.84 t (7.0)
Cna-1	166.5		166.3		166.8		166.3	
2	118.9	6.66 d (16.0)	118.5	6.58 d (16.0)	118.9	6.66 d (16.0)	118.3	6.58 d (16.0)
3	146.7	7.83 d (16.0)	145.2	7.85 d (16.0)	145.7	7.86 d (16.0)	145.3	7.85 d (16.0)
1'	134.7		135.3		135.0		135.3	
2' and 6'	128.5	7.36 m	128.6	7.43 m	128.8	7.42 m	128.4	7.43 m
3' and 5'	129.3	7.30 m	128.9	7.32 m	129.6	7.33 m	129.1	7.33 m
4'	130.3	7.30 m	130.8	7.32 m	131.1	7.33 m	131.0	7.33 m
Dodeca-1	174.0		173.4		173.4			
2	34.4	2.32 *	34.2	2.48 m	34.9	2.34 *		

12	14.7	0.87 *	14.1	0.83 t (7.0)	14.6	0.86 *		
Mba-1	176.6							
2	41.7	2.46 m						
2-CH_3	16.7	1.23 d (7.0)						
4	12.1	0.86 t (7.0)						
Bu-1			175.8	7.32 m	174.8		175.8	
2			34.0	2.30 m	34.8	2.38 t (7.8)	34.0	2.26 m
4			14.1	0.83 t (7.0)	14.6	0.86 *	14.1	0.84 t (7.0)
Tetradeca-1							173.4	
2							34.2	2.53
14							14.1	0.84 t (7.0)

Chemical shifts (δ) are in ppm relative to TMS. The spin coupling (*J*) is given in parentheses (Hz). Chemical shifts marked with an asterisk (*) indicate overlapped signals. Spin-coupled patterns are designated as follows: br s = broad singlet, d = doublet, t = triplet, m = multiplet, q = quartet. Abbreviations: Glc = glucose; Rha = rhamnose; Ag = 11-hydroxyhexadecanoyl; Mba = 2*S*-methylbutanoyl; Cna = *trans*-cinnamoyl; Bu = butyryl; Dodeca = *n*-dodecanoyl; Tetradeca= *n*-tetradecanoyl.

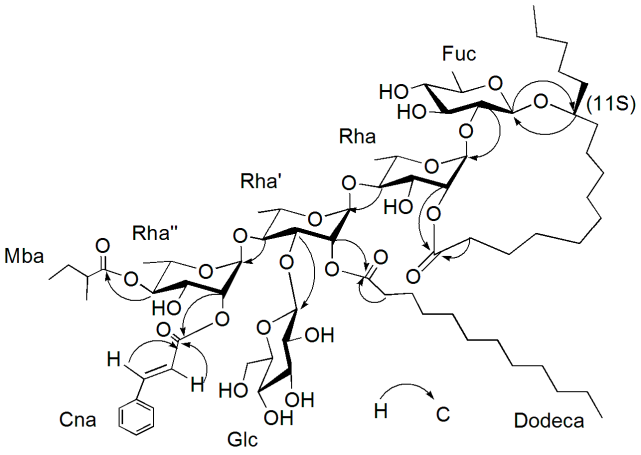

Figure S1. Key HMBC correlations from H to C for Acutacoside F (**1**).

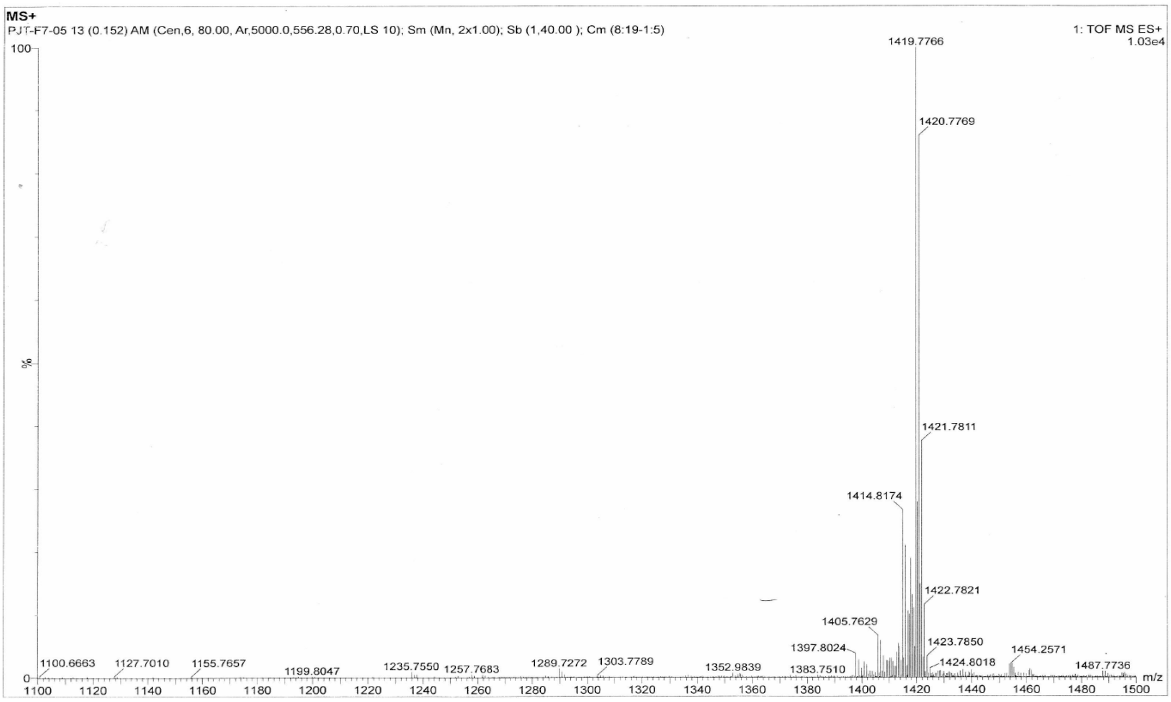

Figure S2. The HR-TOF-MS spectrum of compound **1**

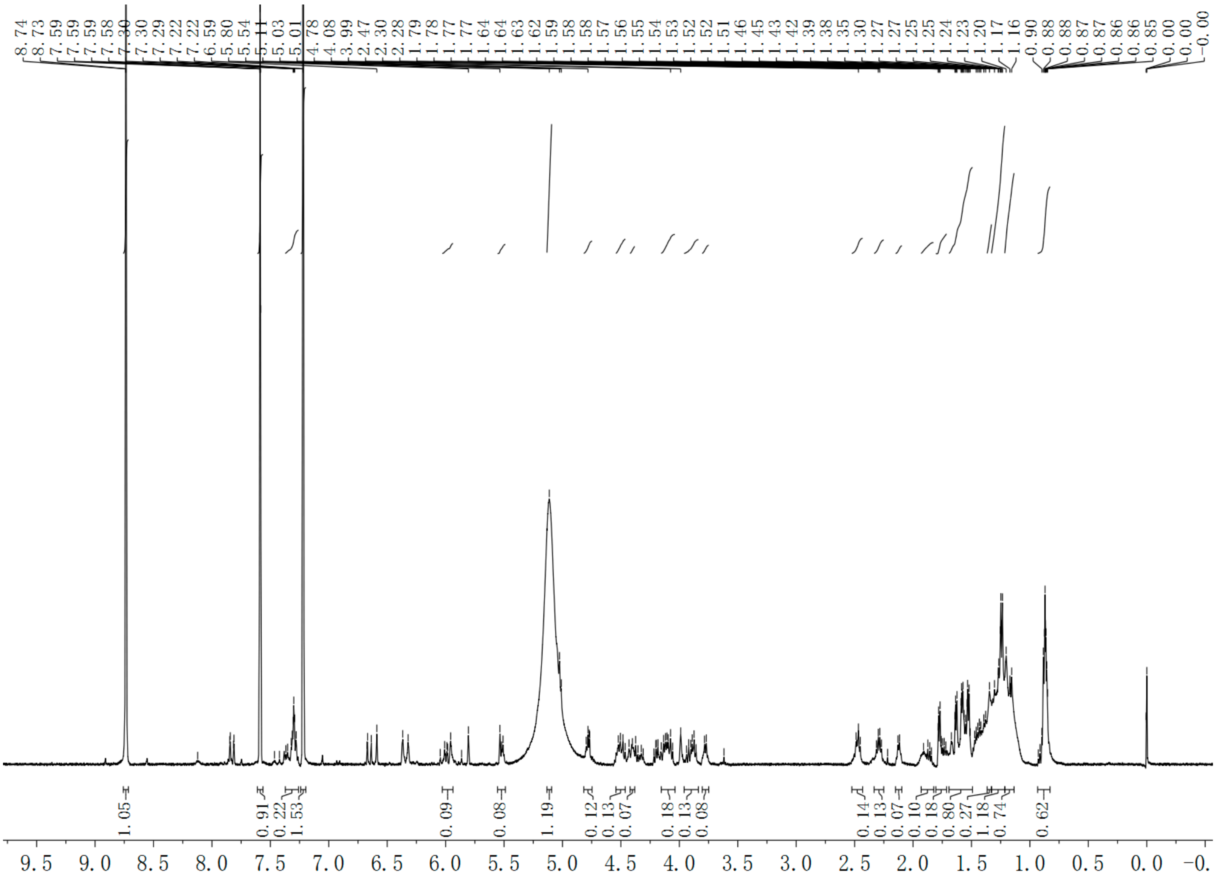

Figure S3. The ^{1}H-NMR spectrum of compound **1**

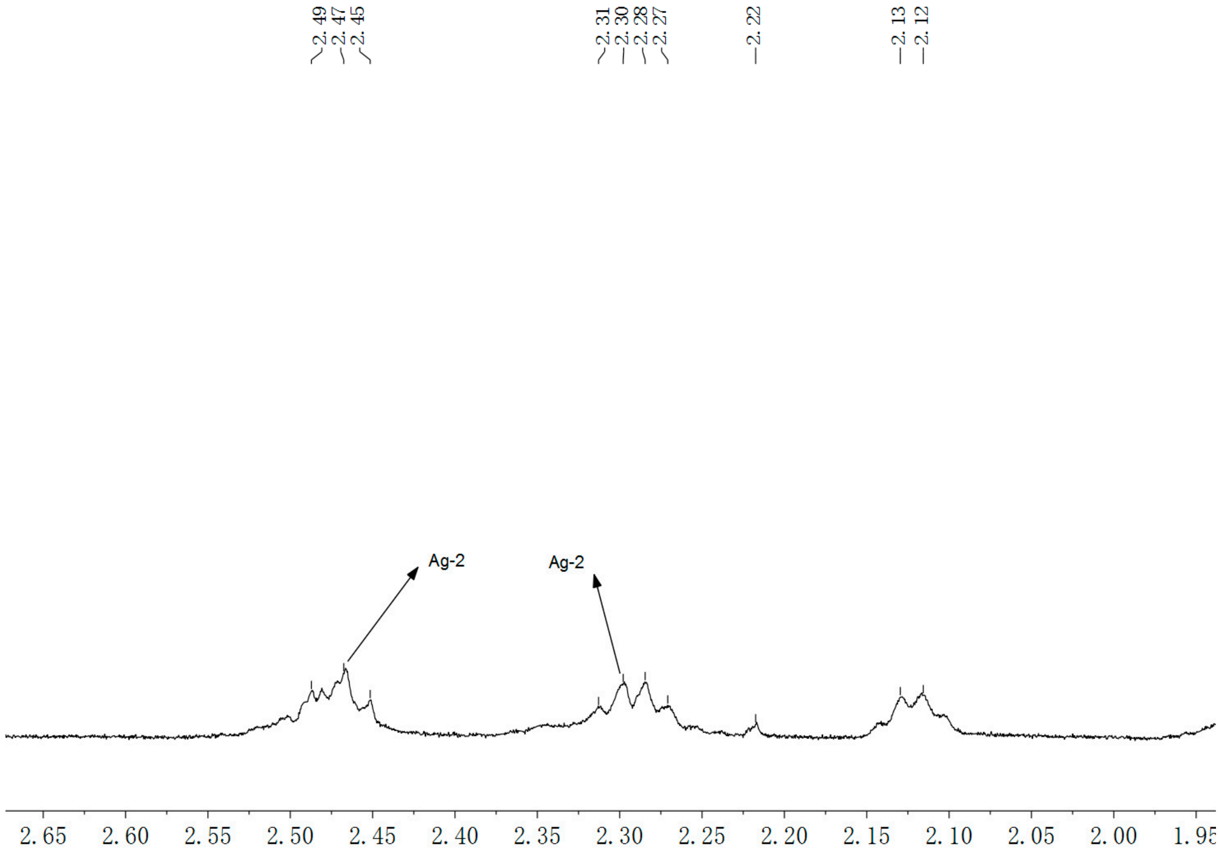

Figure S3-1. The ^{1}H-NMR spectrum of compound **1**

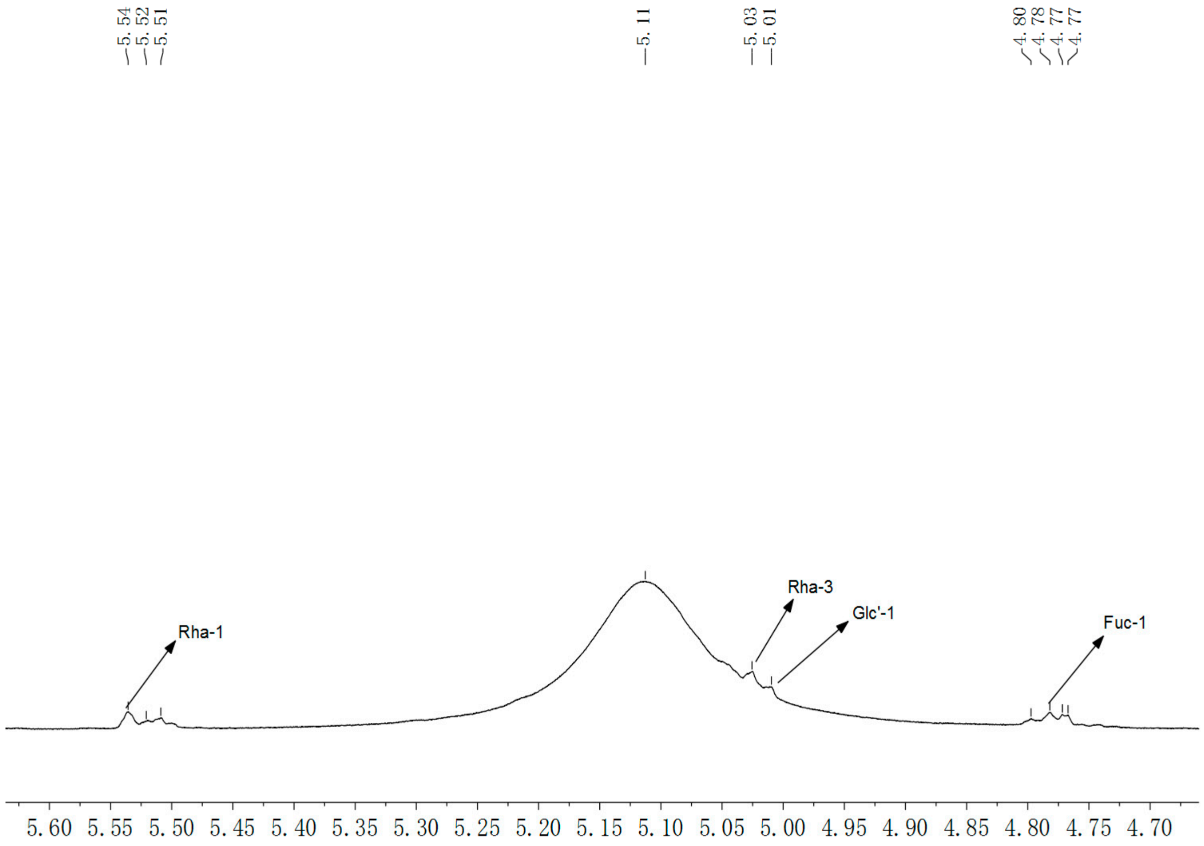

Figure S3-2. The ^{1}H-NMR spectrum of compound **1**

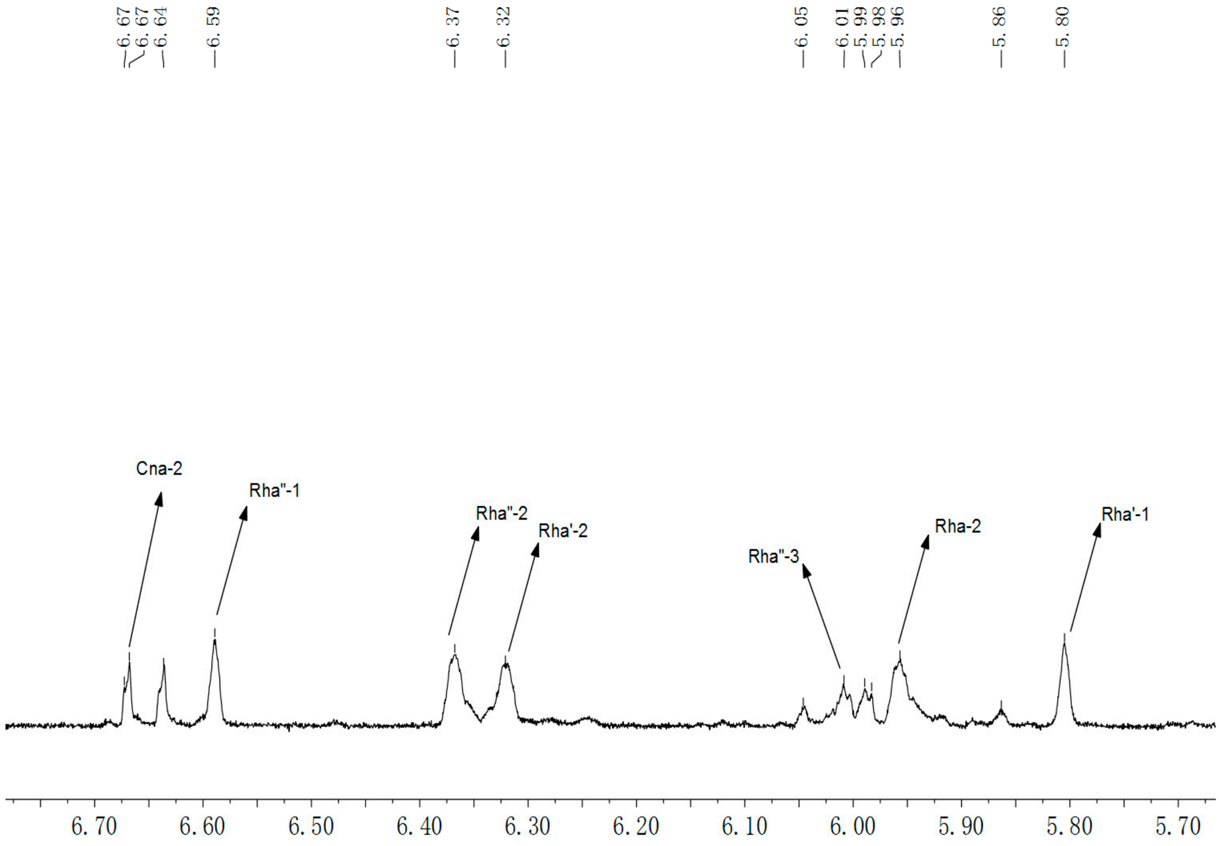

Figure S3-3. The ^{1}H-NMR spectrum of compound **1**

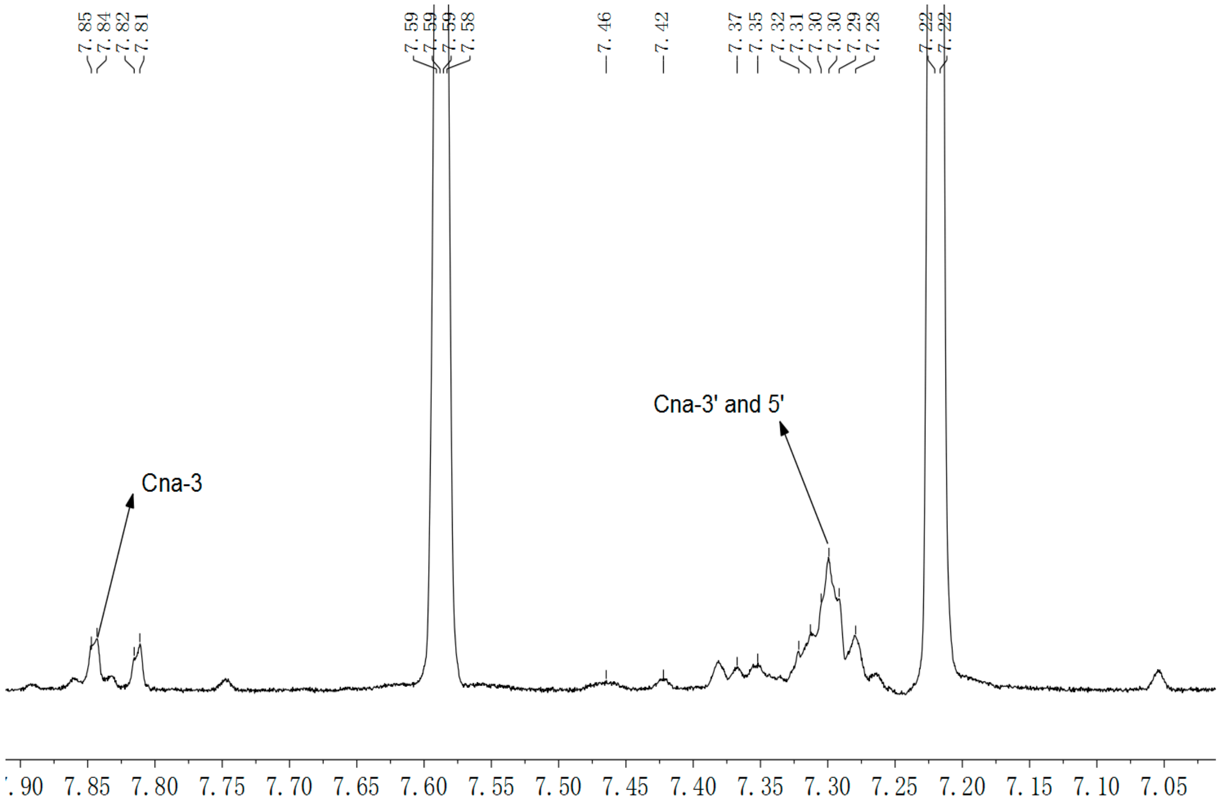

Figure S3-4. The ^{1}H-NMR spectrum of compound **1**

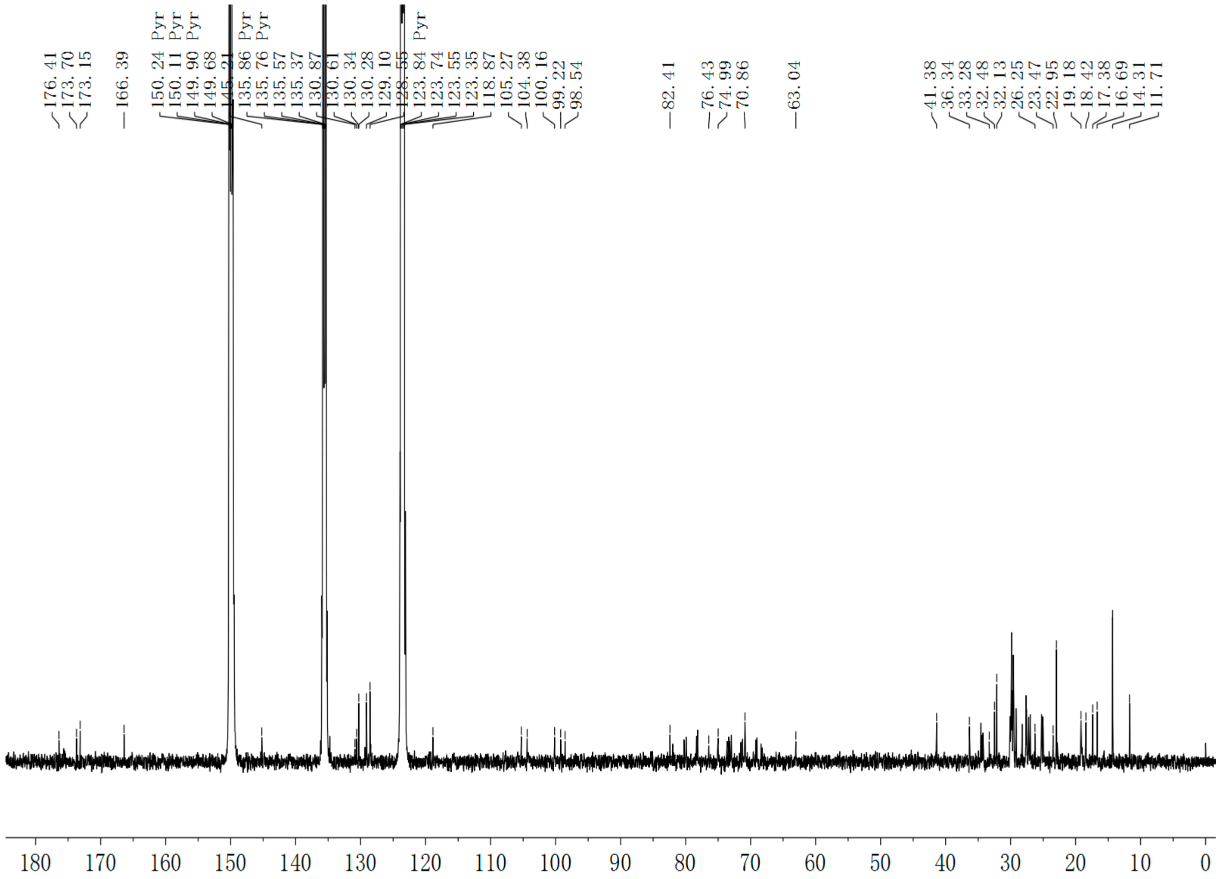

Figure S4. The ^{13}C-NMR spectrum of compound 1

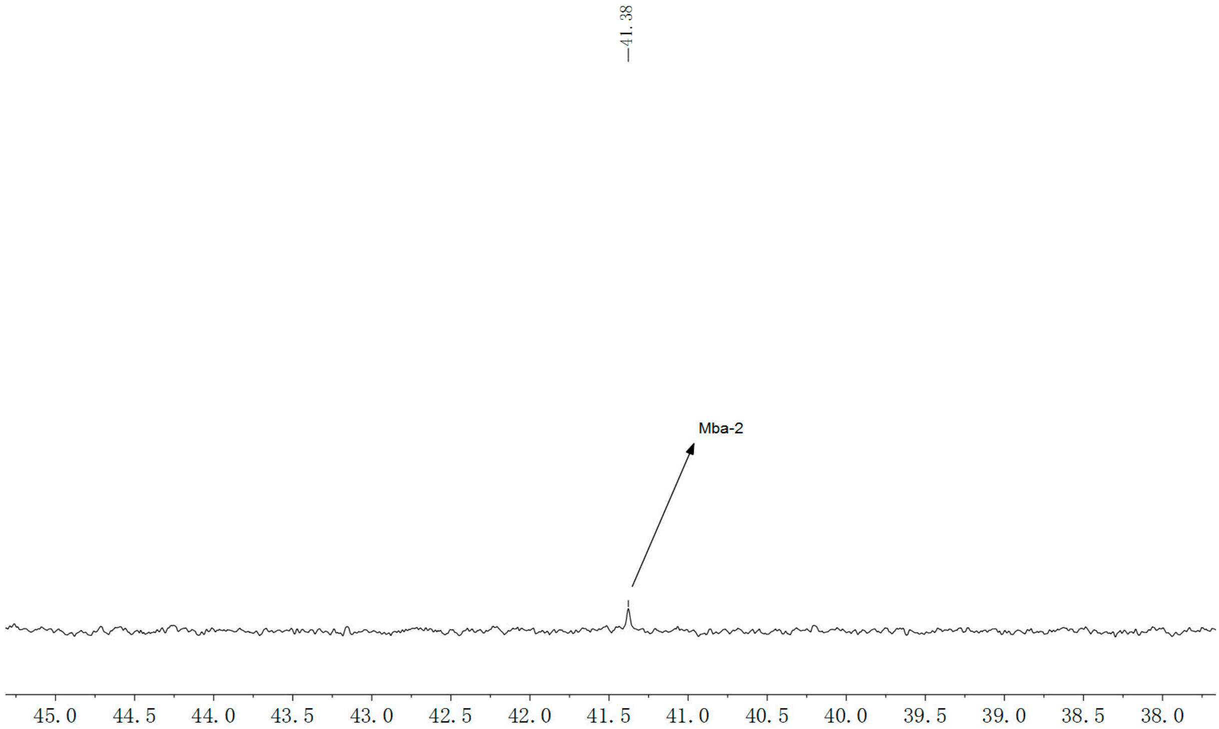

Figure S4-1. The ^{13}C-NMR spectrum of compound 1

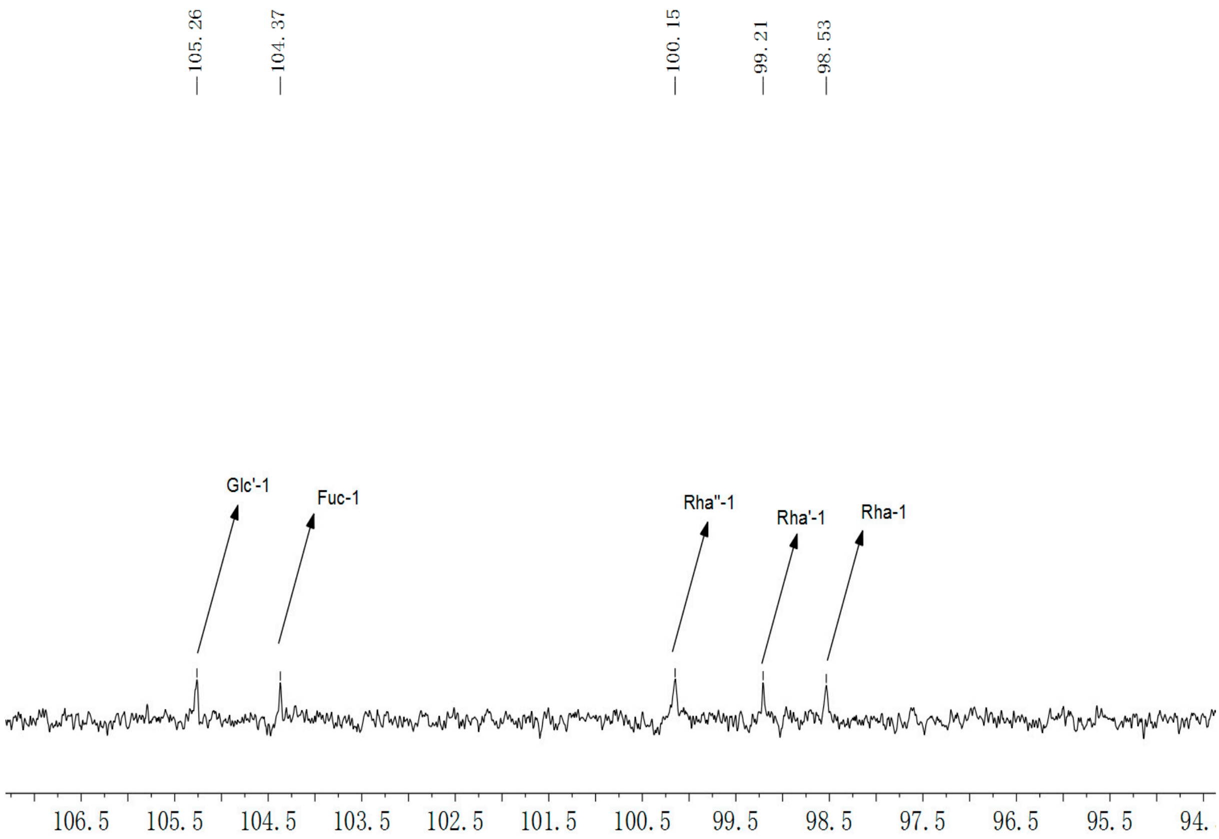

Figure S4-2. The ^{13}C-NMR spectrum of compound 1

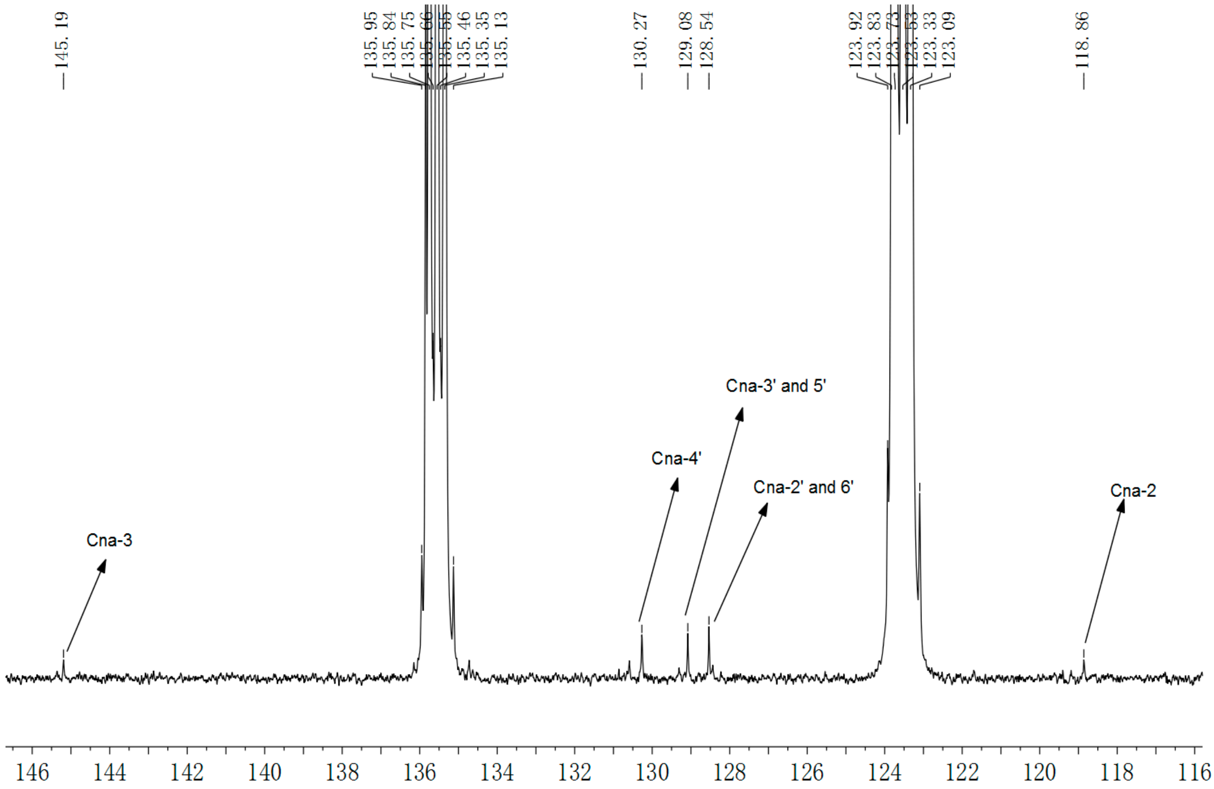

Figure S4-3. The ^{13}C-NMR spectrum of compound 1

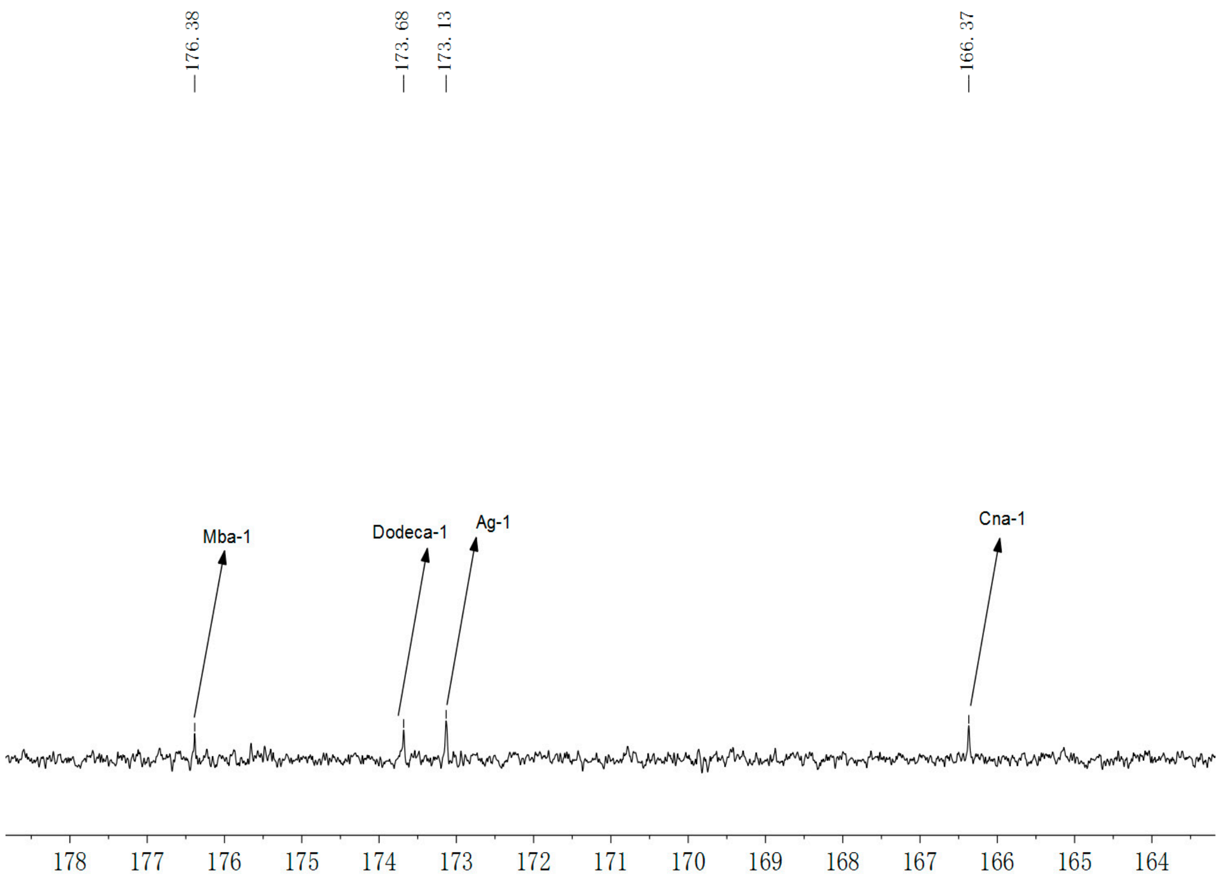

Figure S4-4. The ^{13}C-NMR spectrum of compound 1

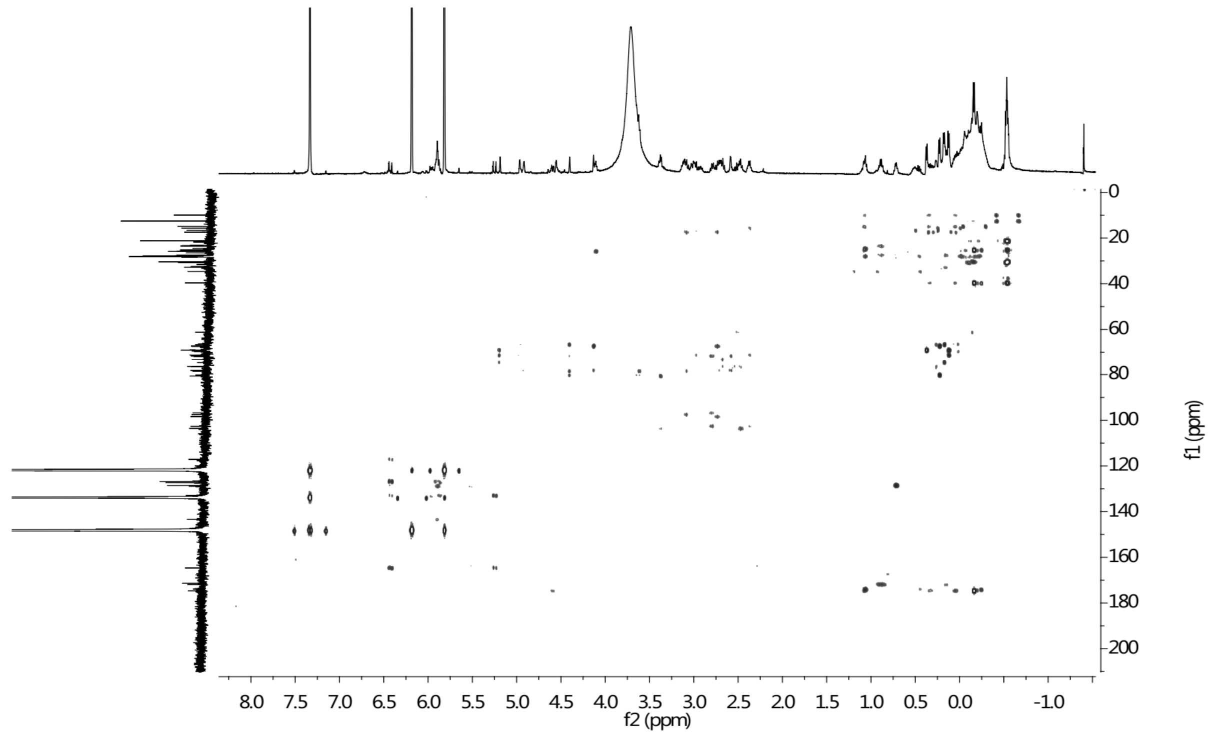

Figure S5. The HMBC spectrum of compound **1**

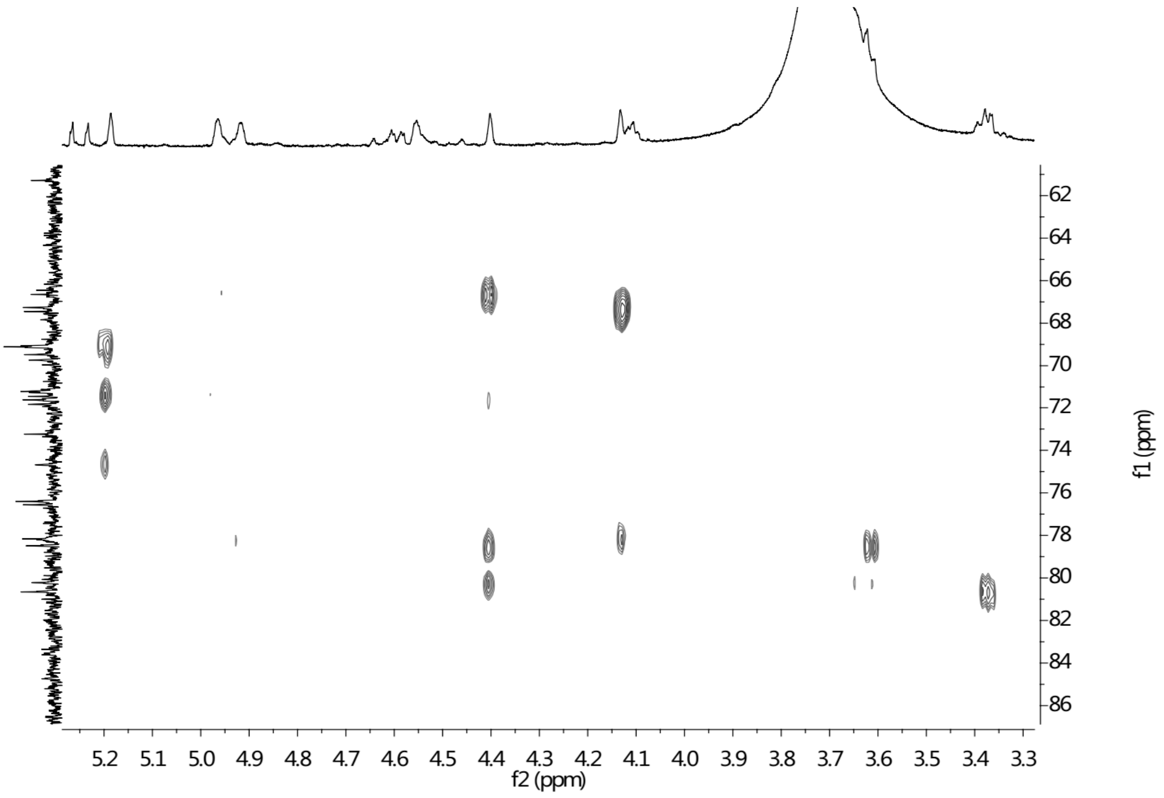

Figure S5-1. The HMBC spectrum of compound **1**

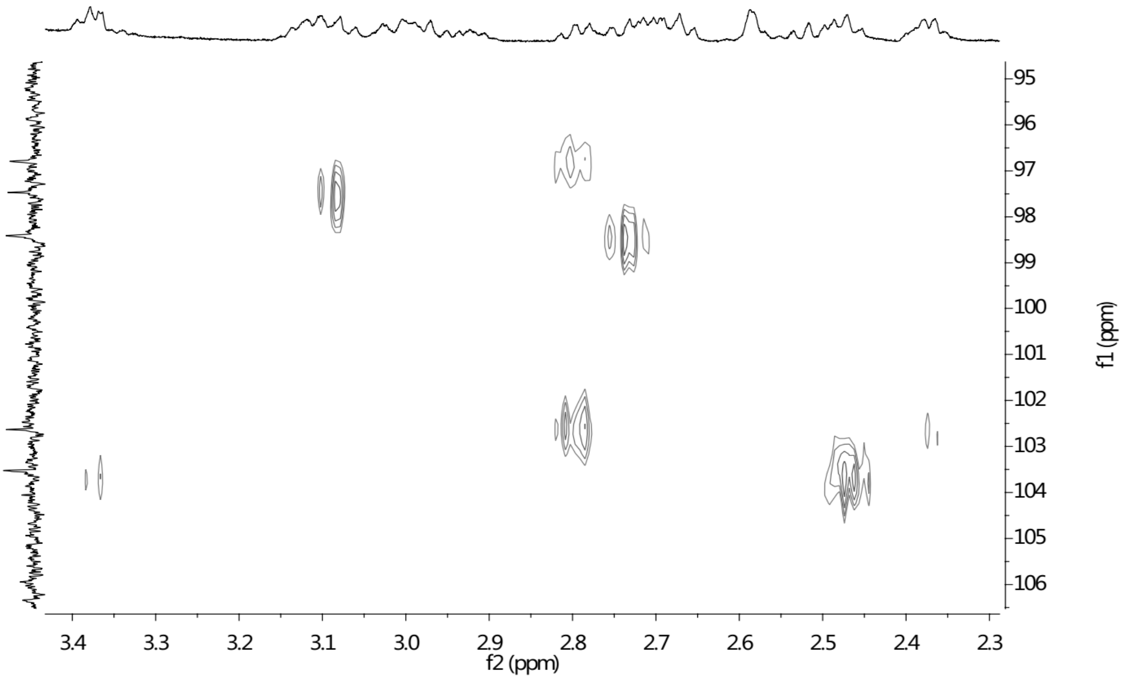

Figure S5-2. The HMBC spectrum of compound **1**

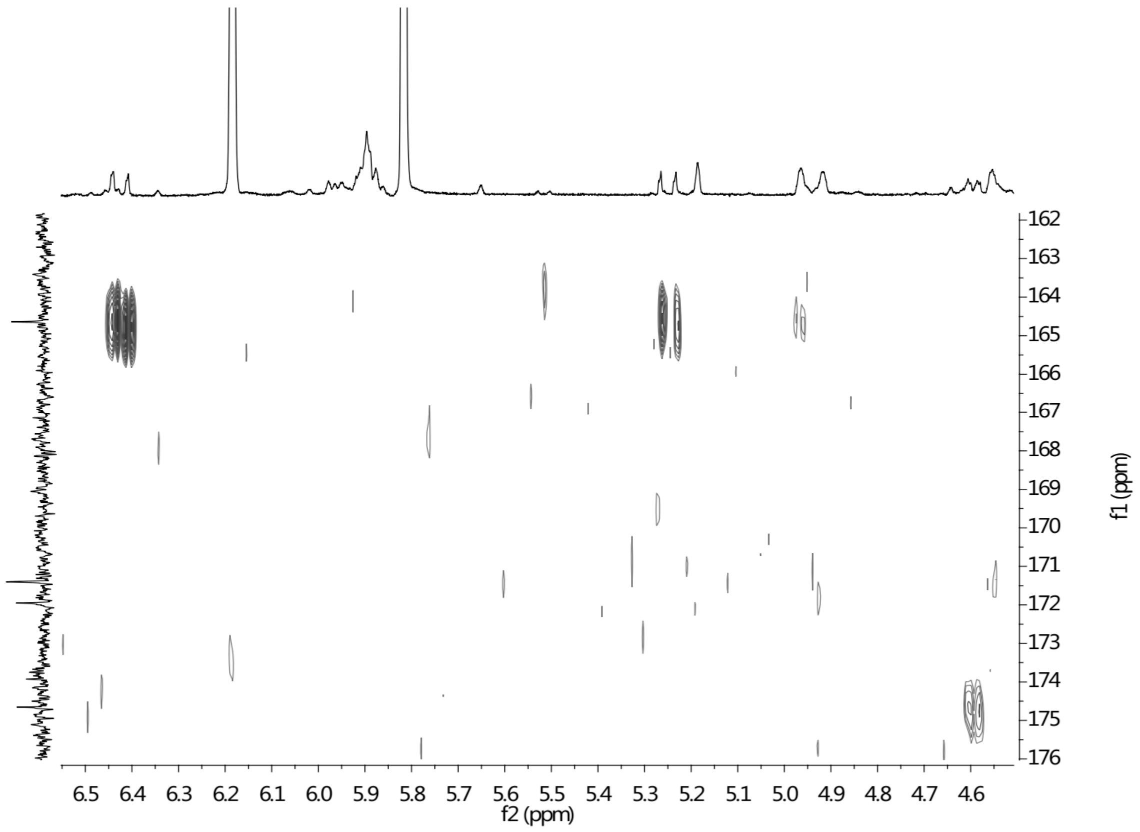

Figure S5-3. The HMBC spectrum of compound **1**

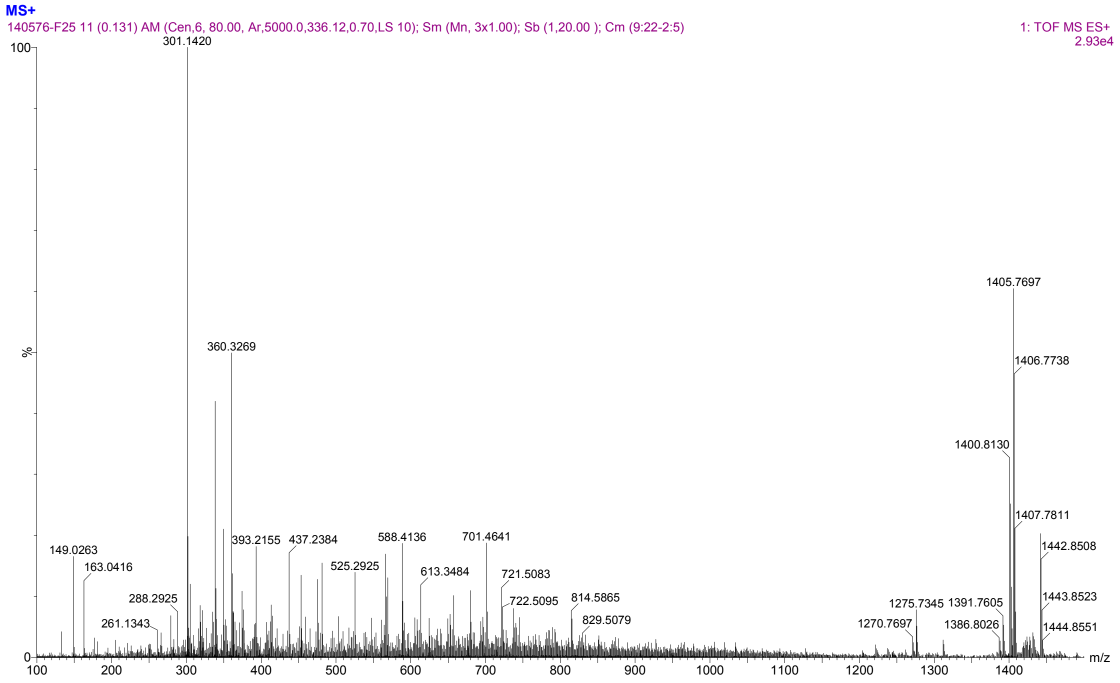

Figure S6. The HR-TOF-MS spectrum of compound **2**

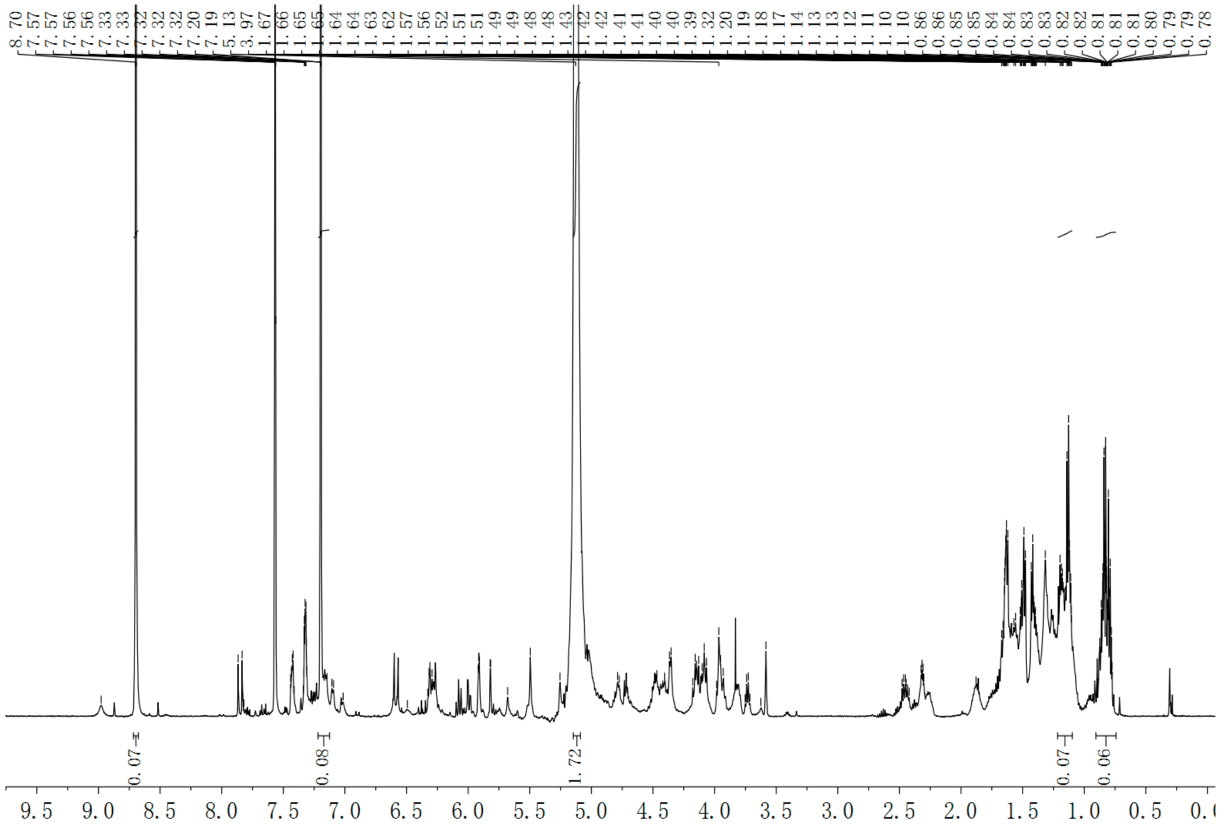

Figure S7. The ^{1}H-NMR spectrum of compound **2**

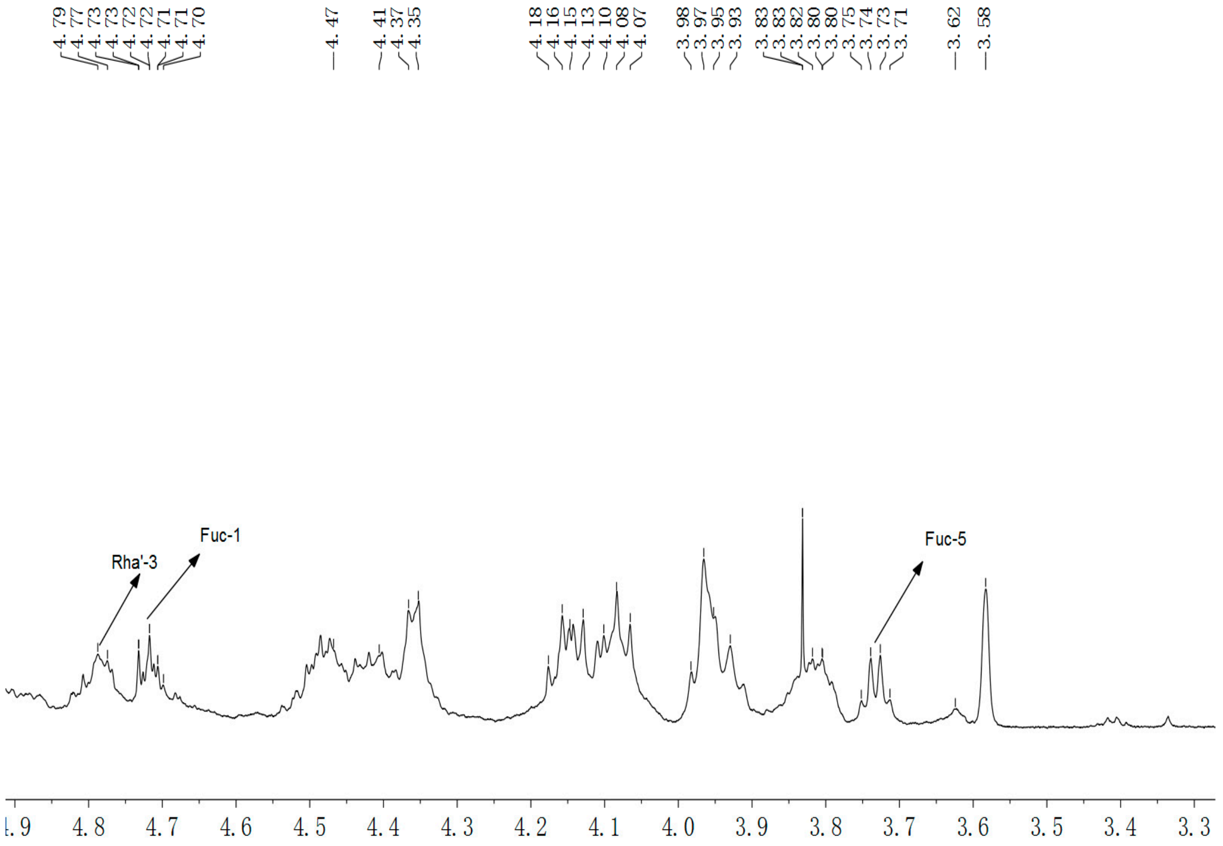

Figure S7-1. The ^{1}H-NMR spectrum of compound **2**

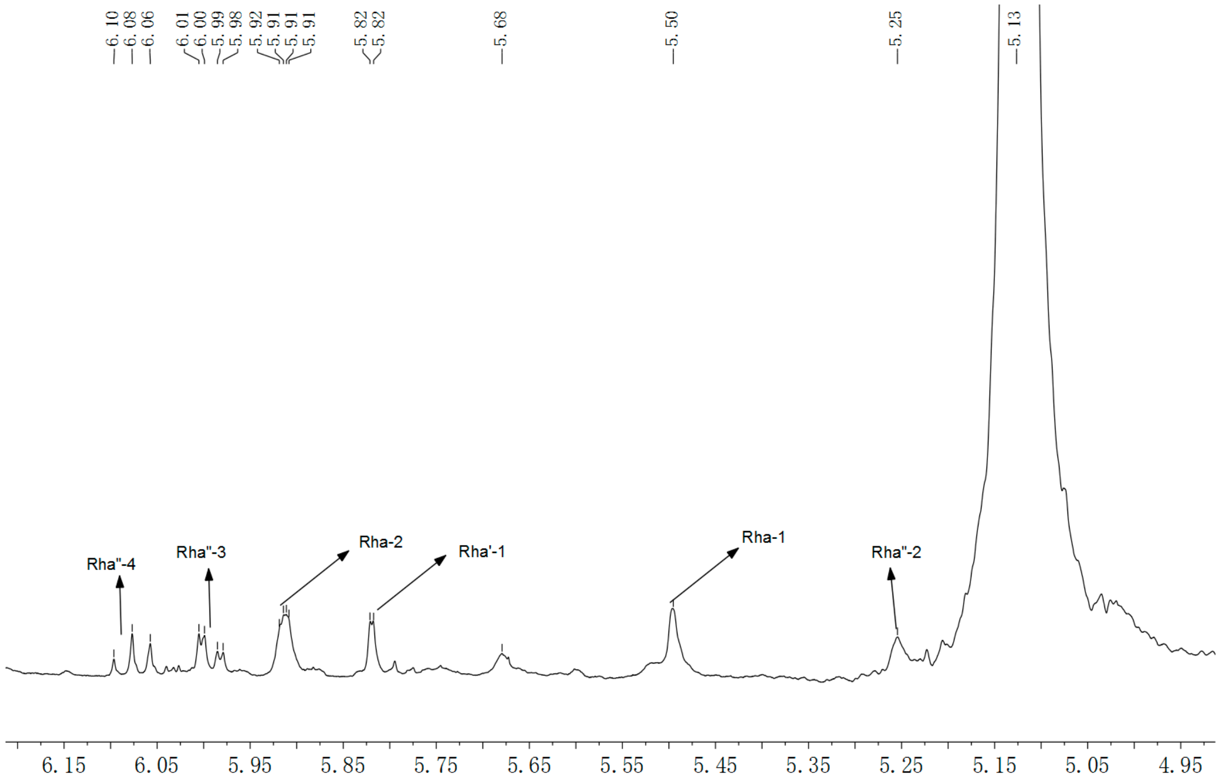

Figure S7-2. The ^{1}H-NMR spectrum of compound **2**

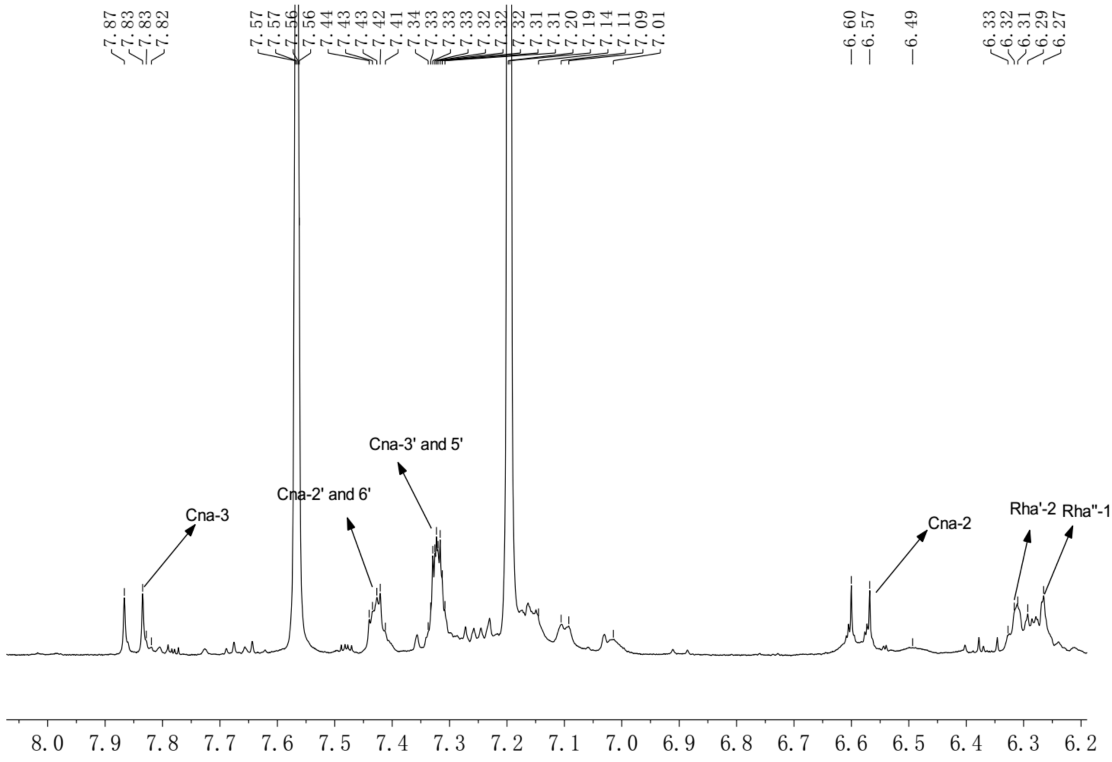

Figure S7-3. The ^{1}H-NMR spectrum of compound **2**

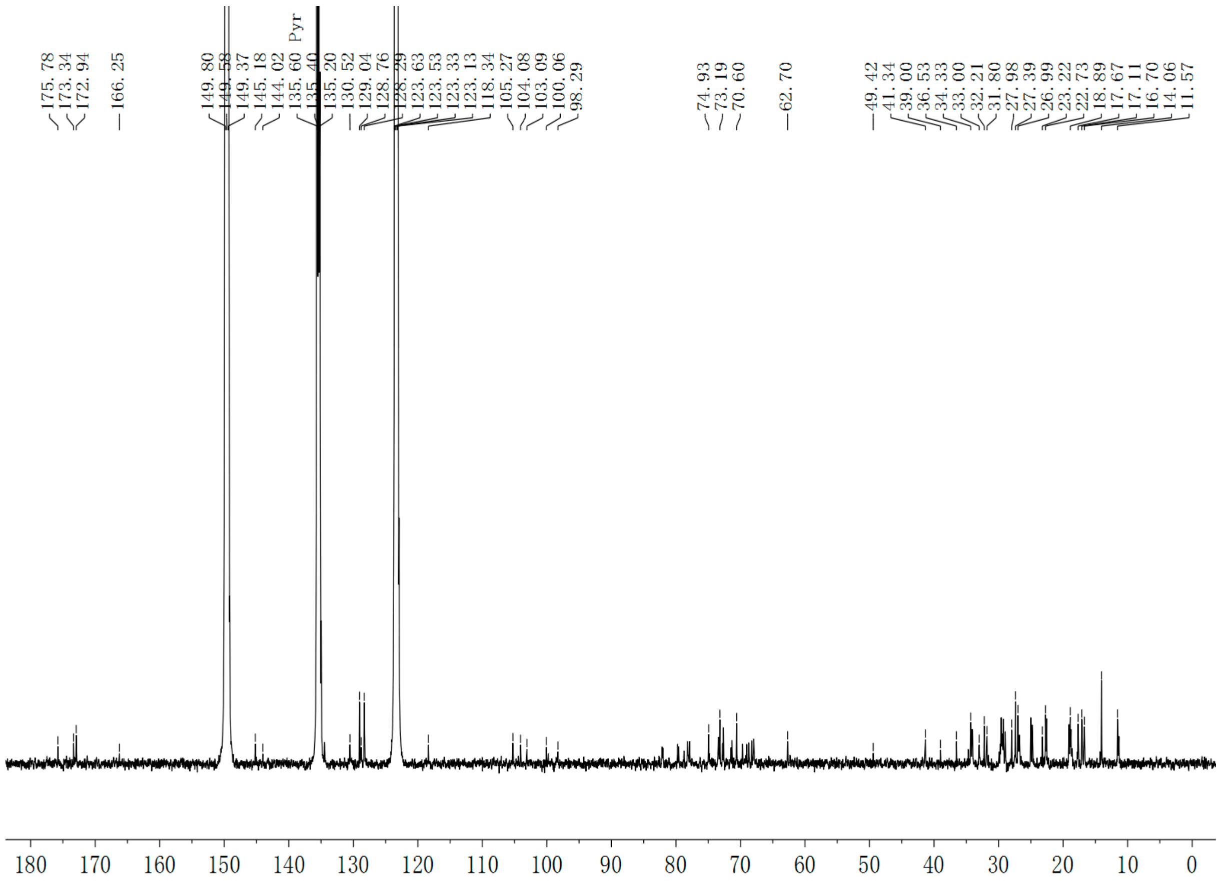

Figure S8. The ^{13}C-NMR spectrum of compound **2**

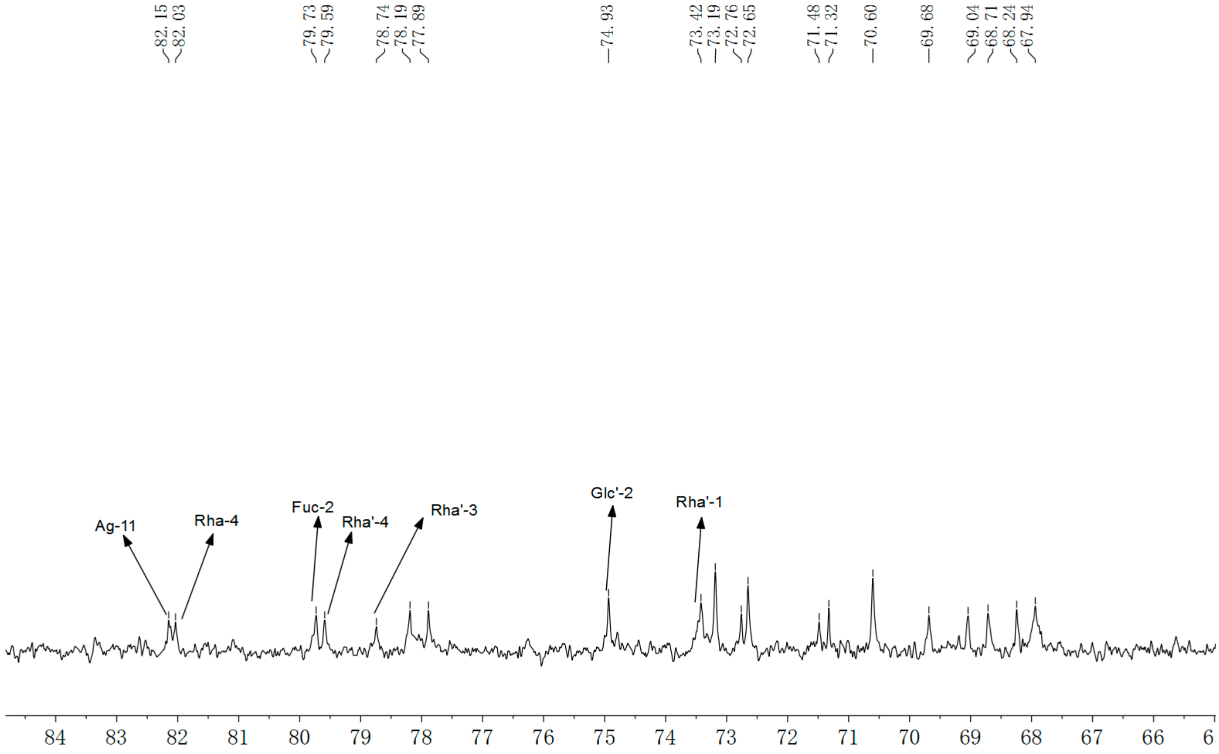

Figure S8-1. The ^{13}C-NMR spectrum of compound **2**

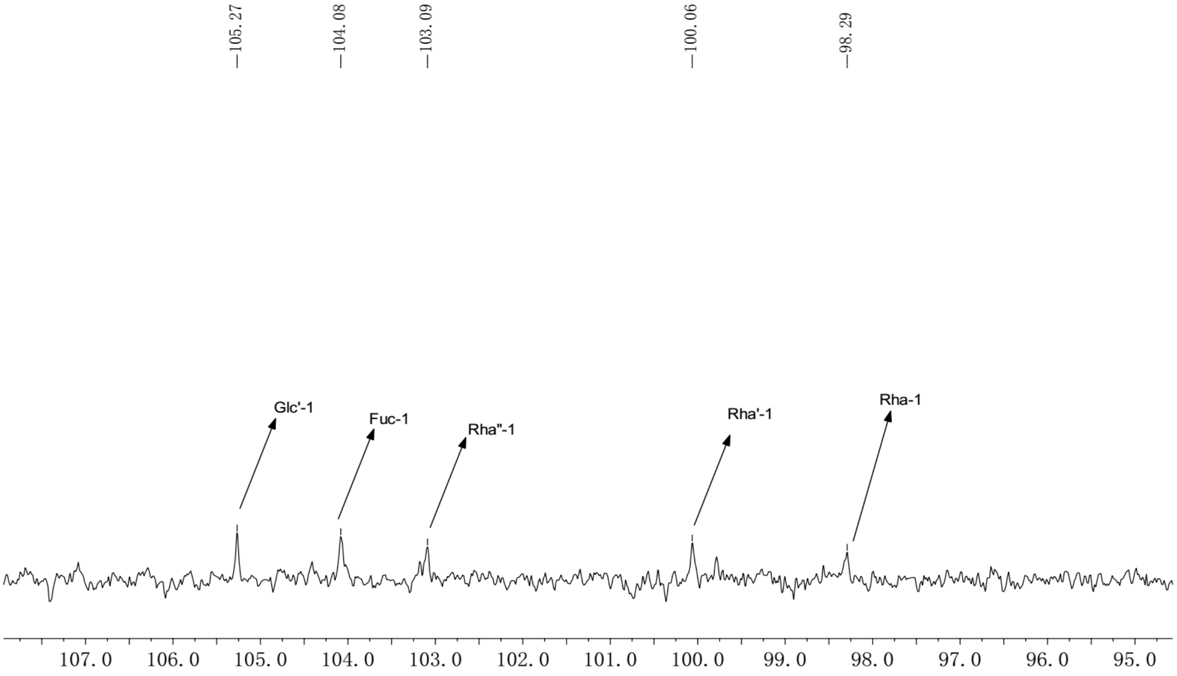

Figure S8-2. The ^{13}C-NMR spectrum of compound **2**

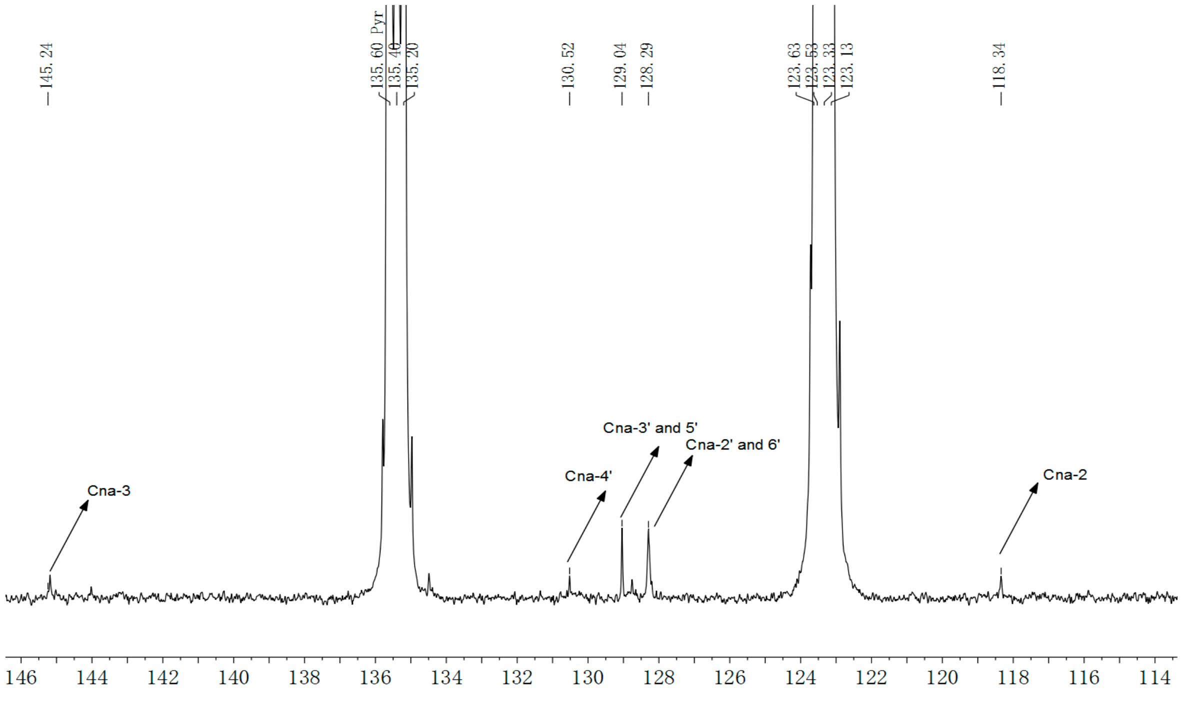

Figure S8-3. The ^{13}C-NMR spectrum of compound **2**

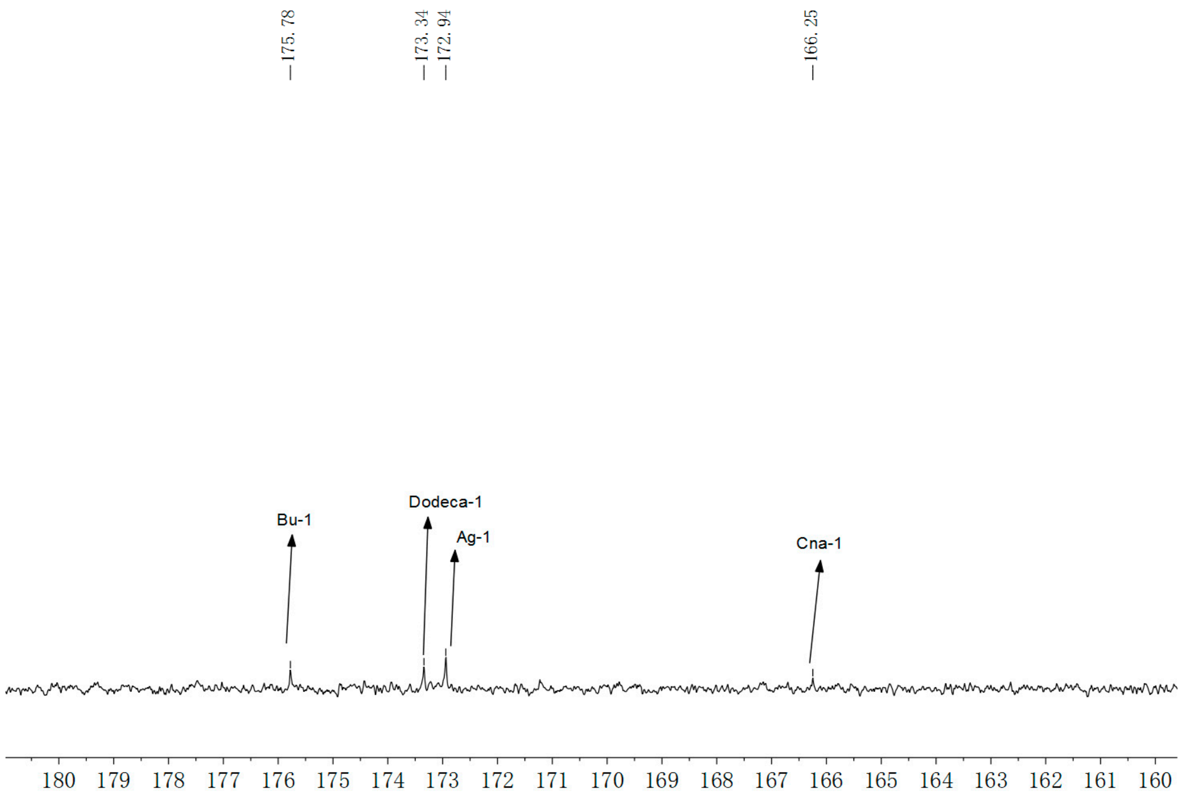

Figure S8-4. The ^{13}C-NMR spectrum of compound **2**

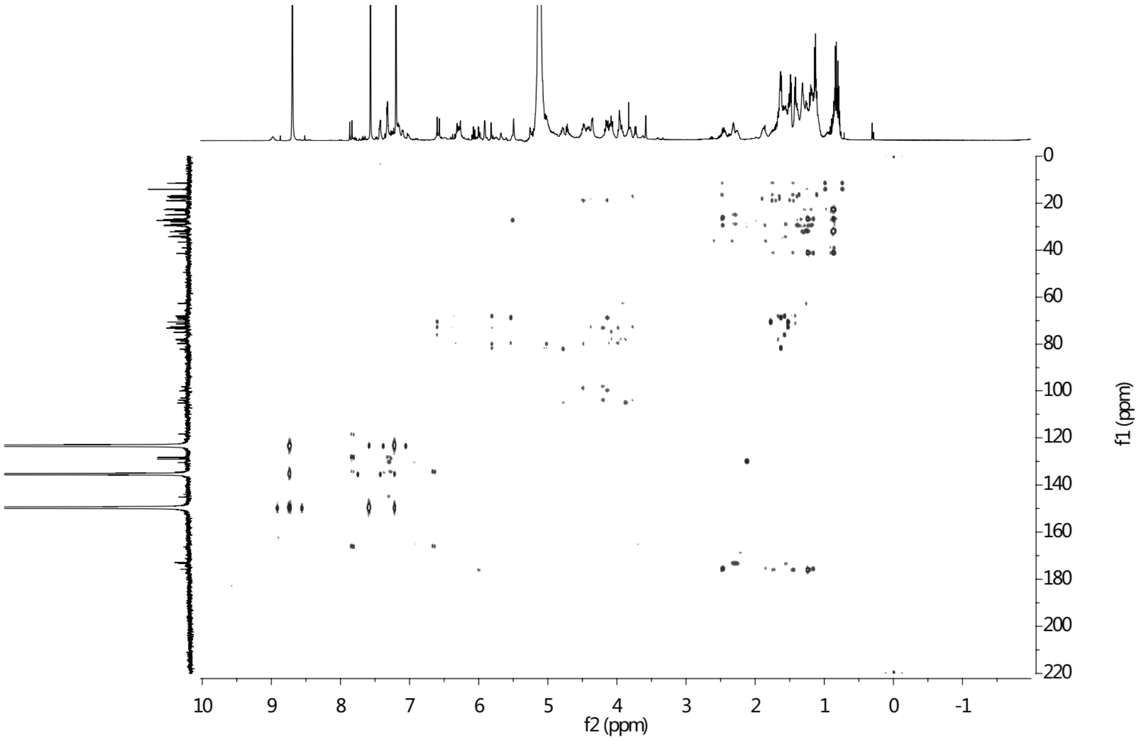

Figure S9. The HMBC spectrum of compound **2**

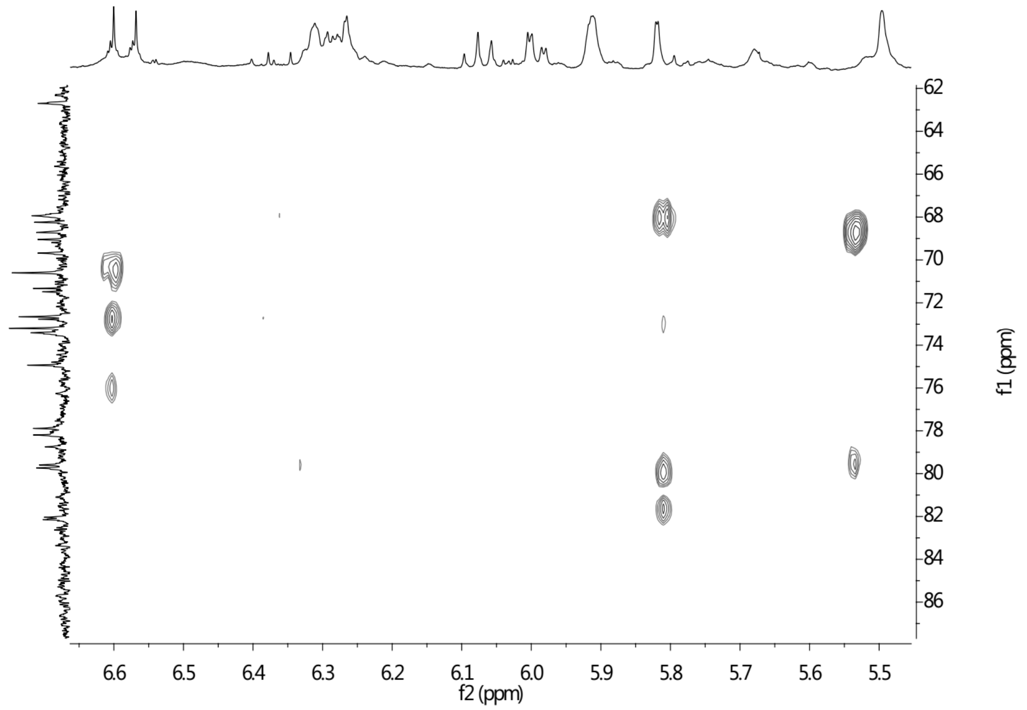

Figure S9-1. The HMBC spectrum of compound **2**

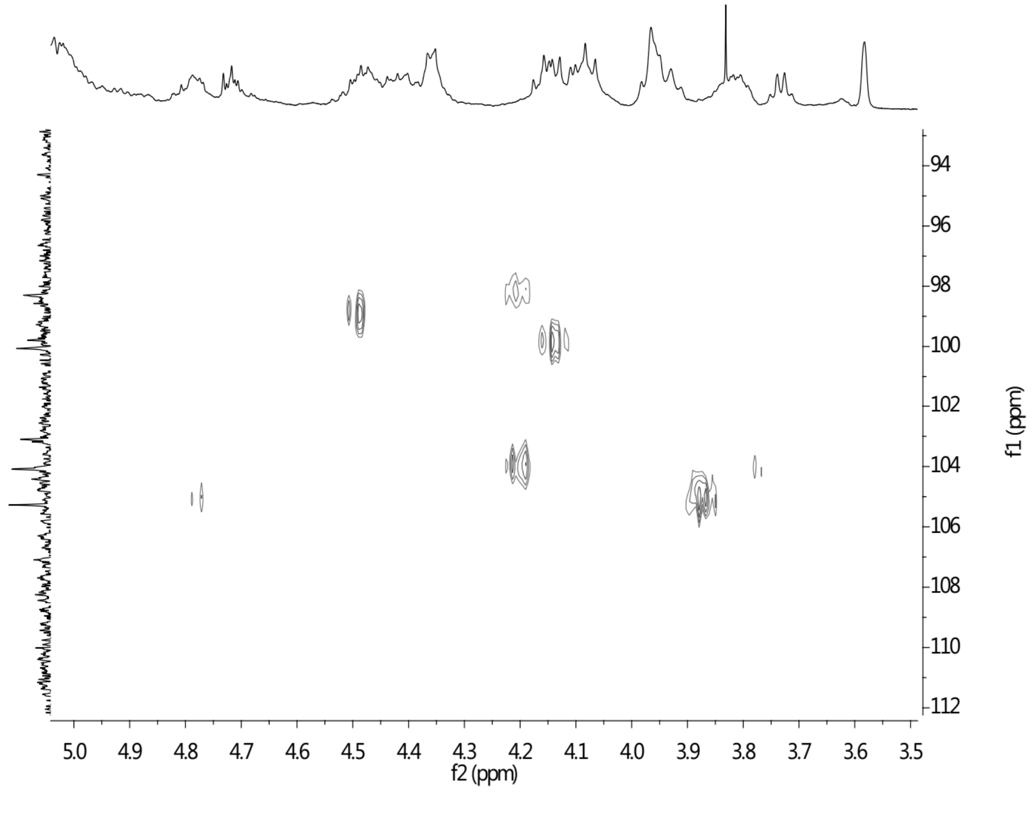

Figure S9-2. The HMBC spectrum of compound **2**

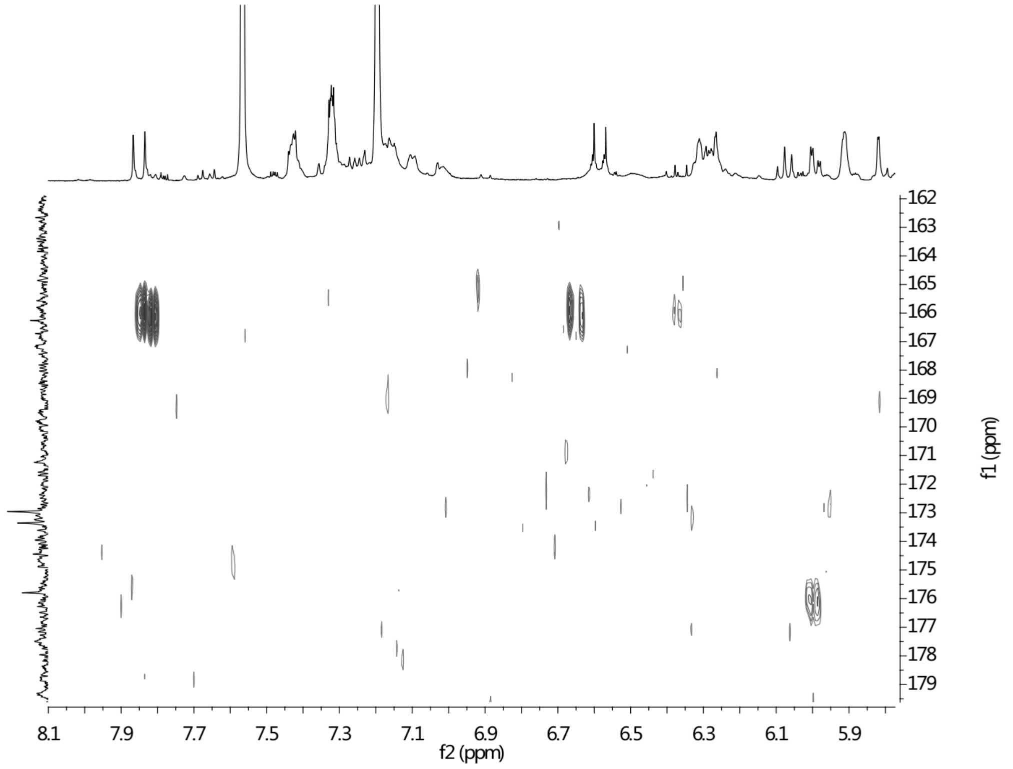

Figure S9-3. The HMBC spectrum of compound **2**

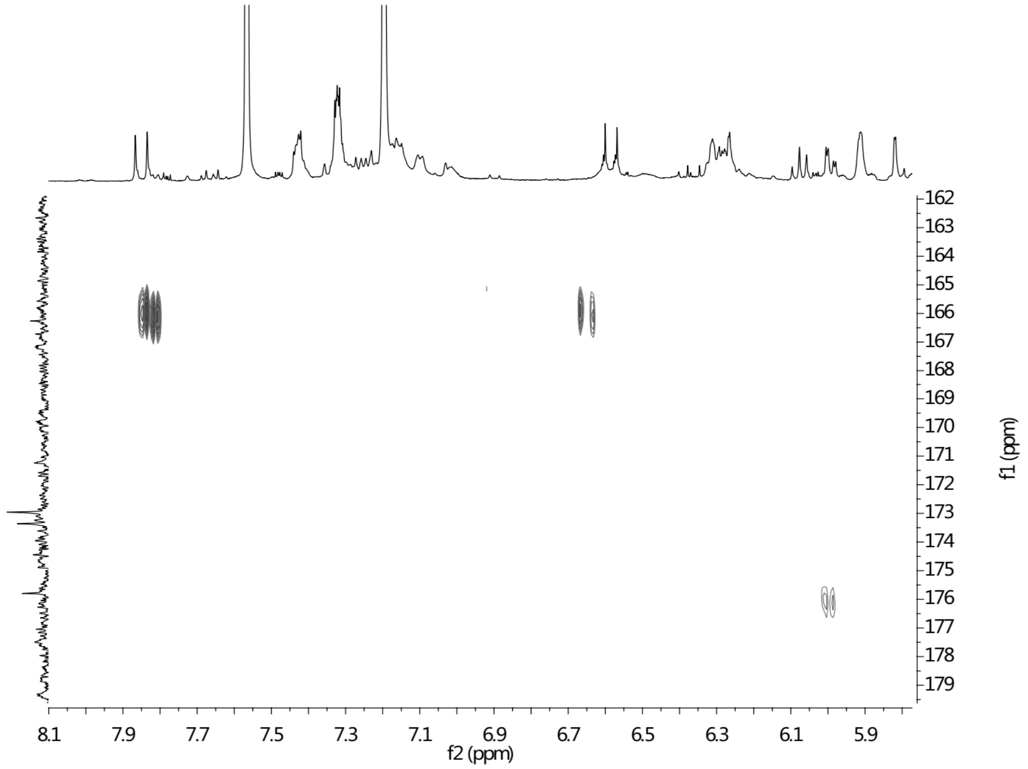

Figure S9-4. The HMBC spectrum of compound **2**

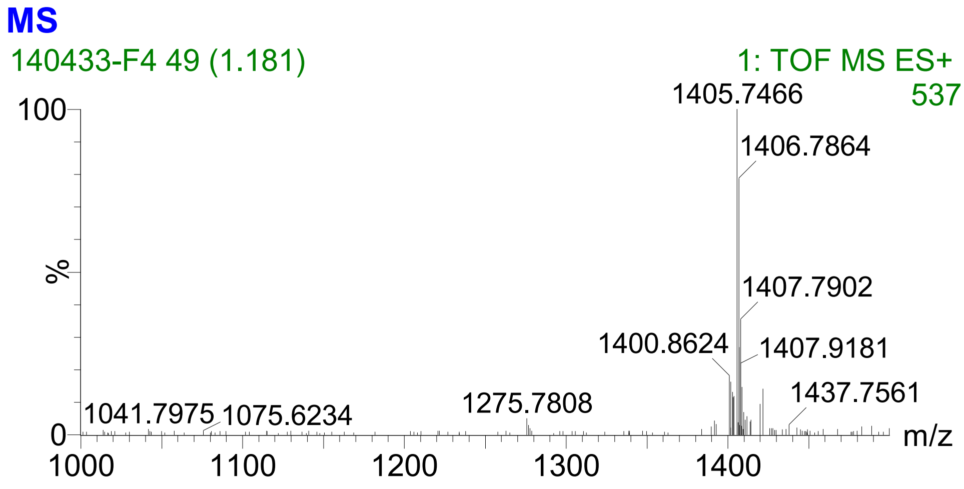

Figure S10. The HR-TOF-MS spectrum of compound **3**

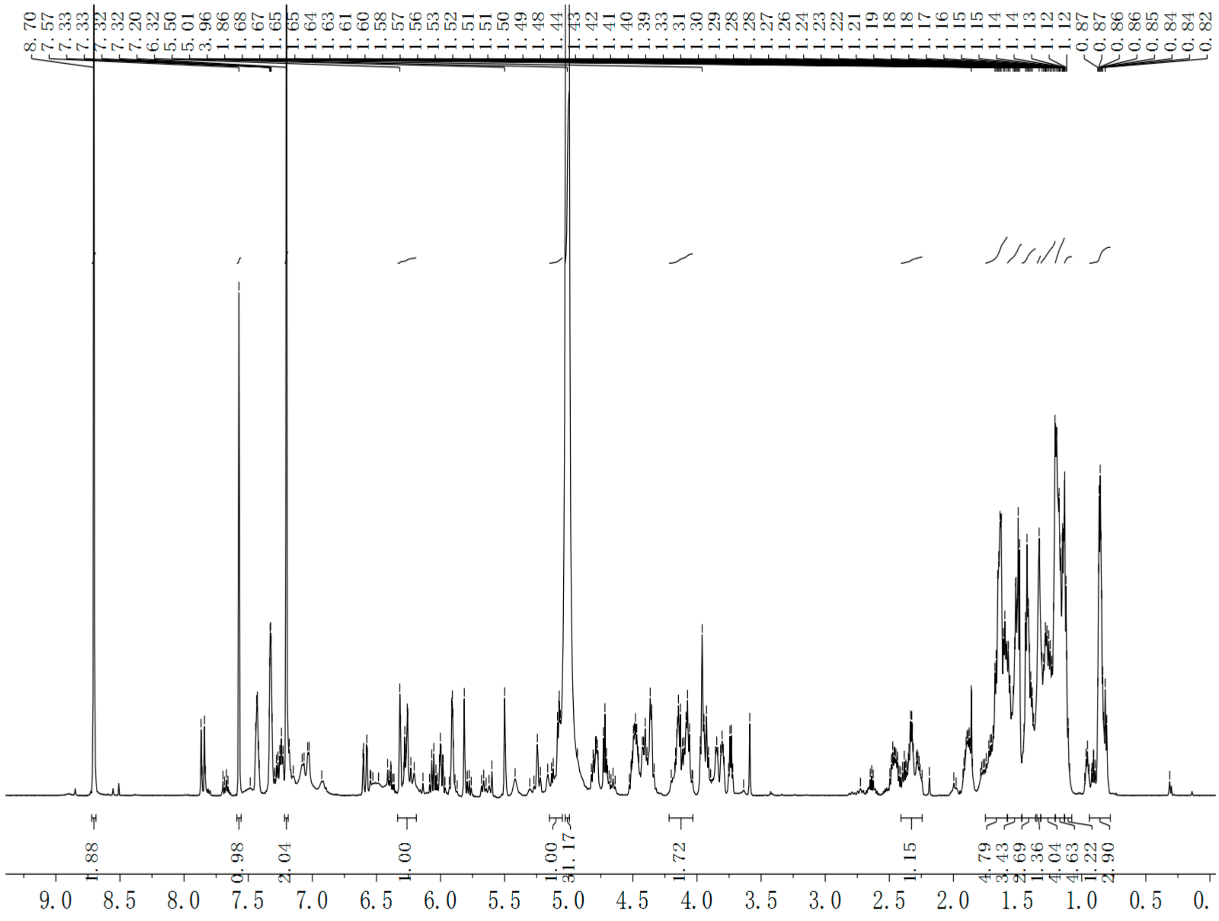

Figure S11. The ^{1}H-NMR spectrum of compound **3**

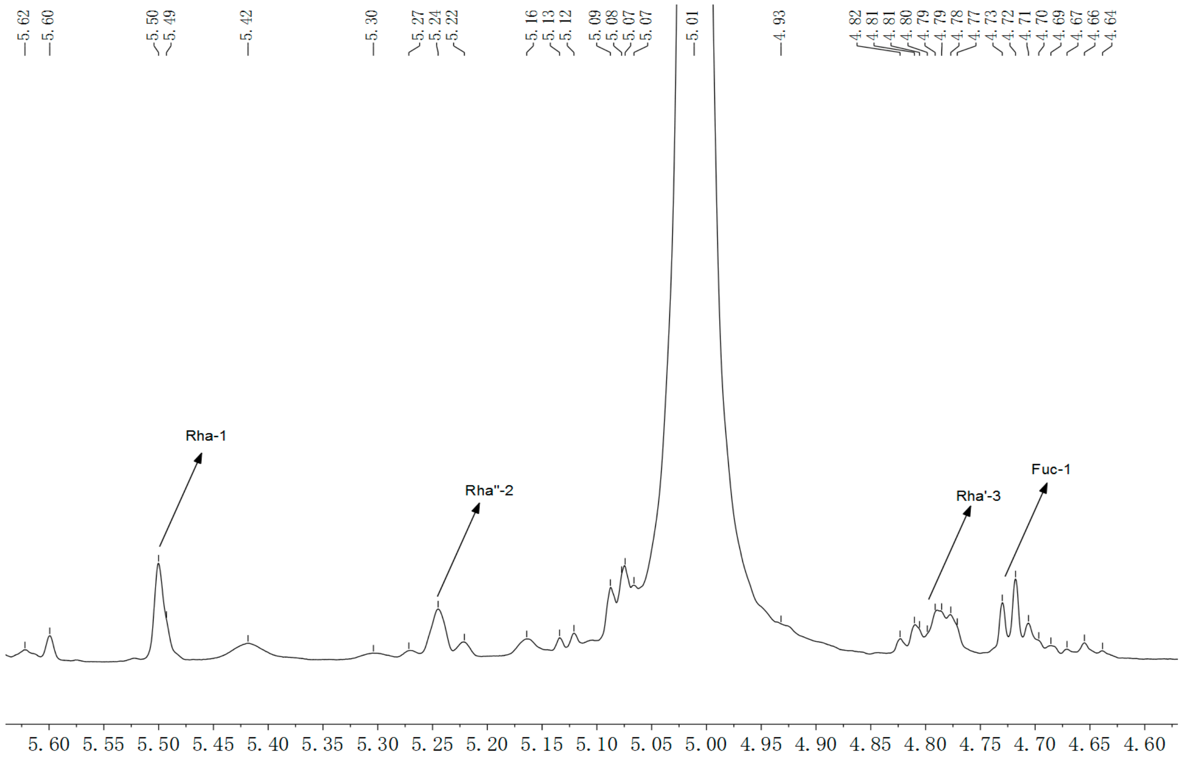

Figure S11-1. The ^{1}H-NMR spectrum of compound **3**

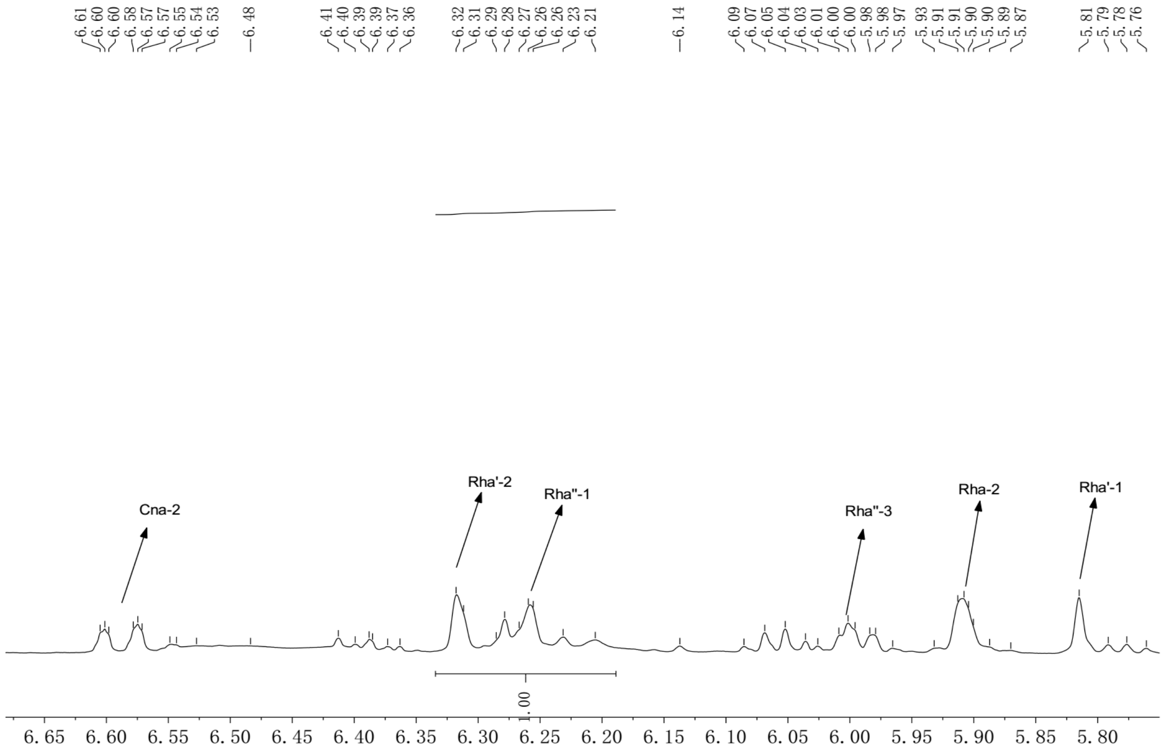

Figure S11-2. The ^{1}H-NMR spectrum of compound **3**

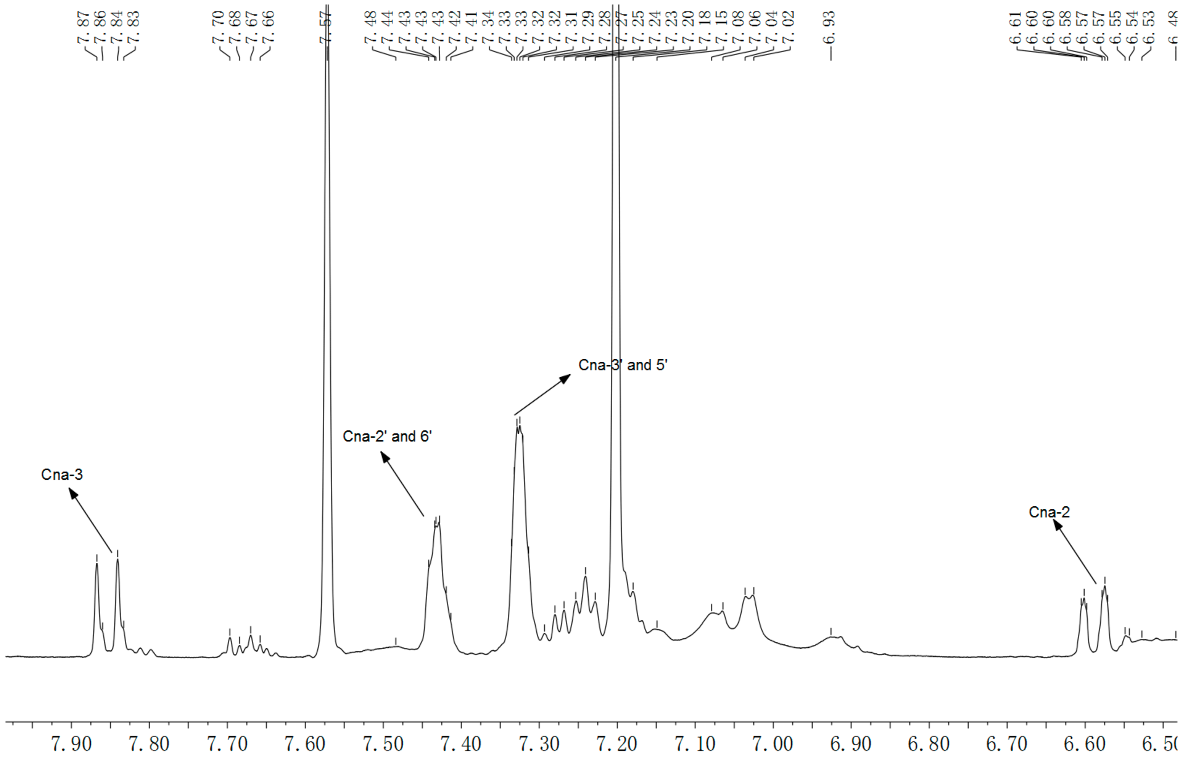

Figure S11-3. The ^{1}H-NMR spectrum of compound **3**

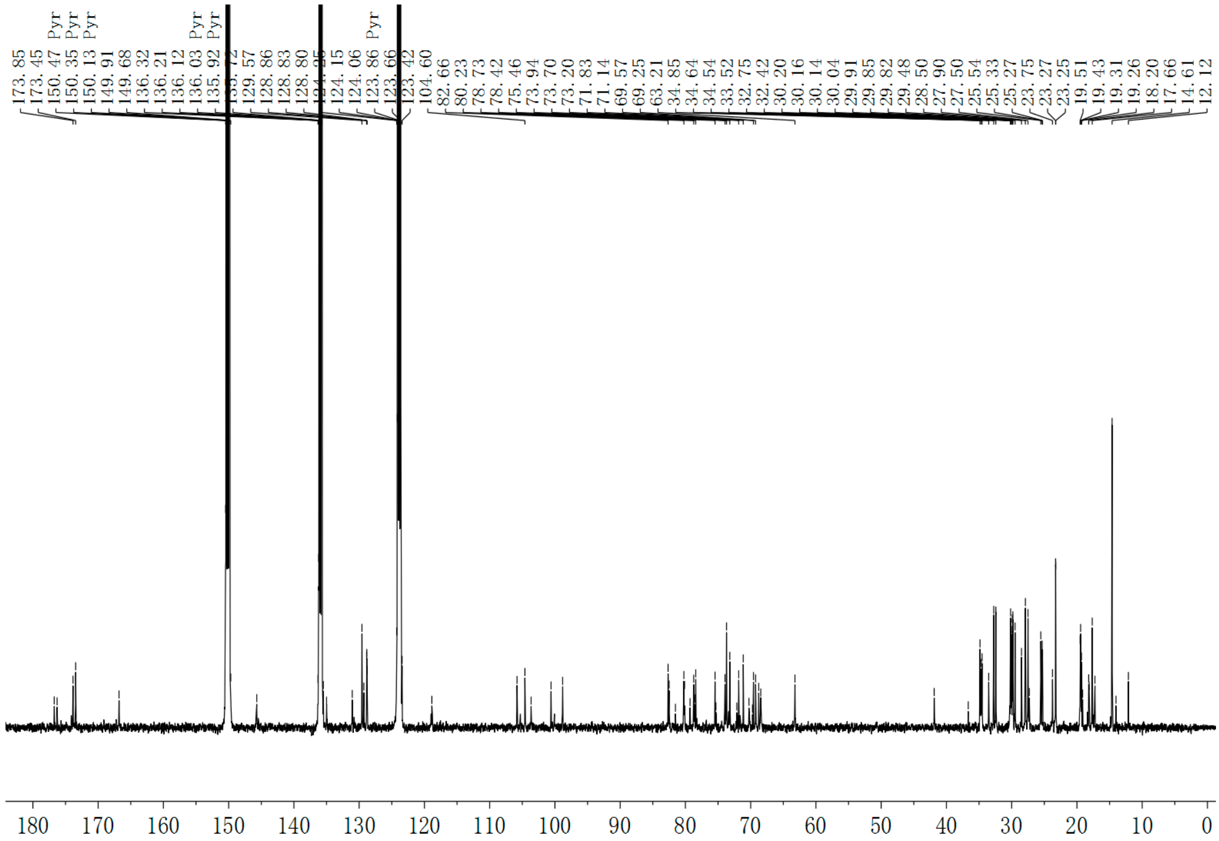

Figure S12. The ^{13}C-NMR spectrum of compound **3**

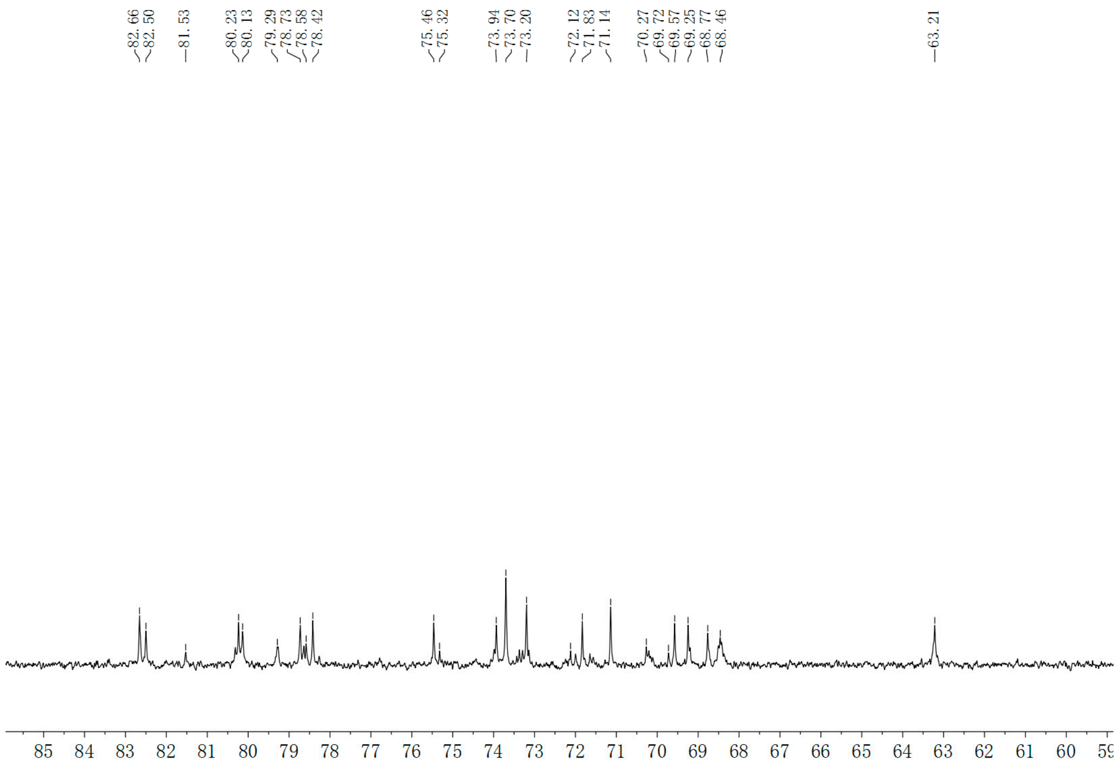

Figure S12-1. The ^{13}C-NMR spectrum of compound **3**

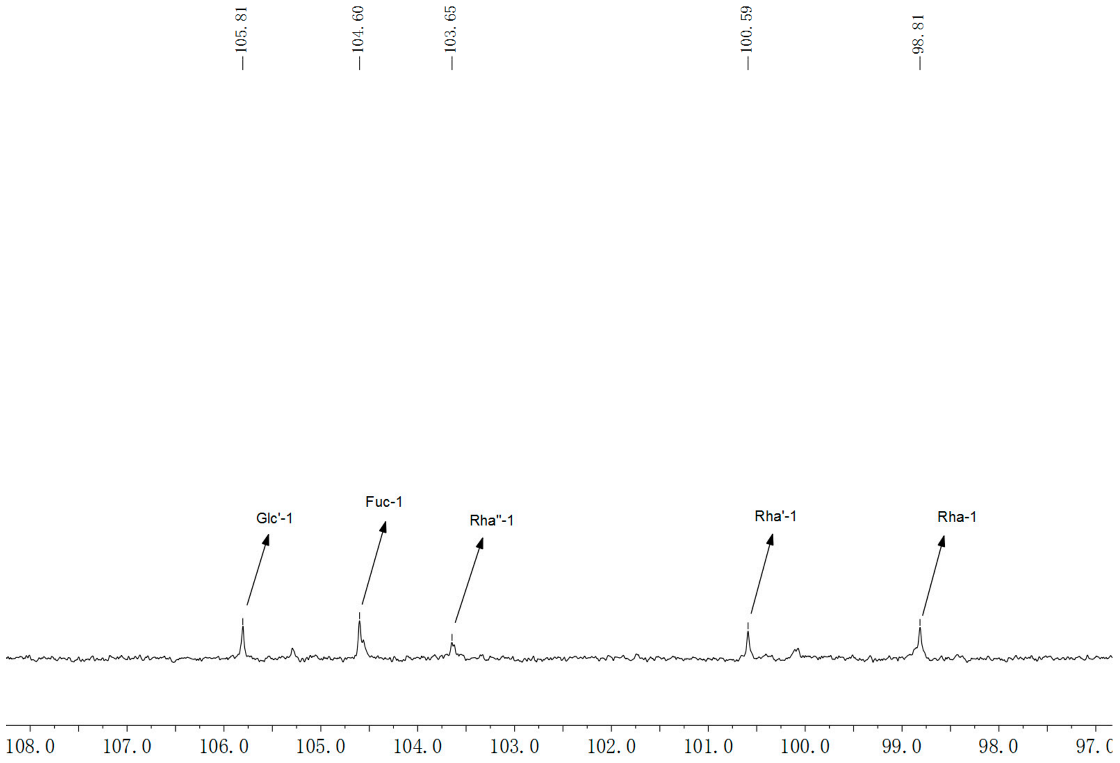

Figure S12-2. The ^{13}C-NMR spectrum of compound **3**

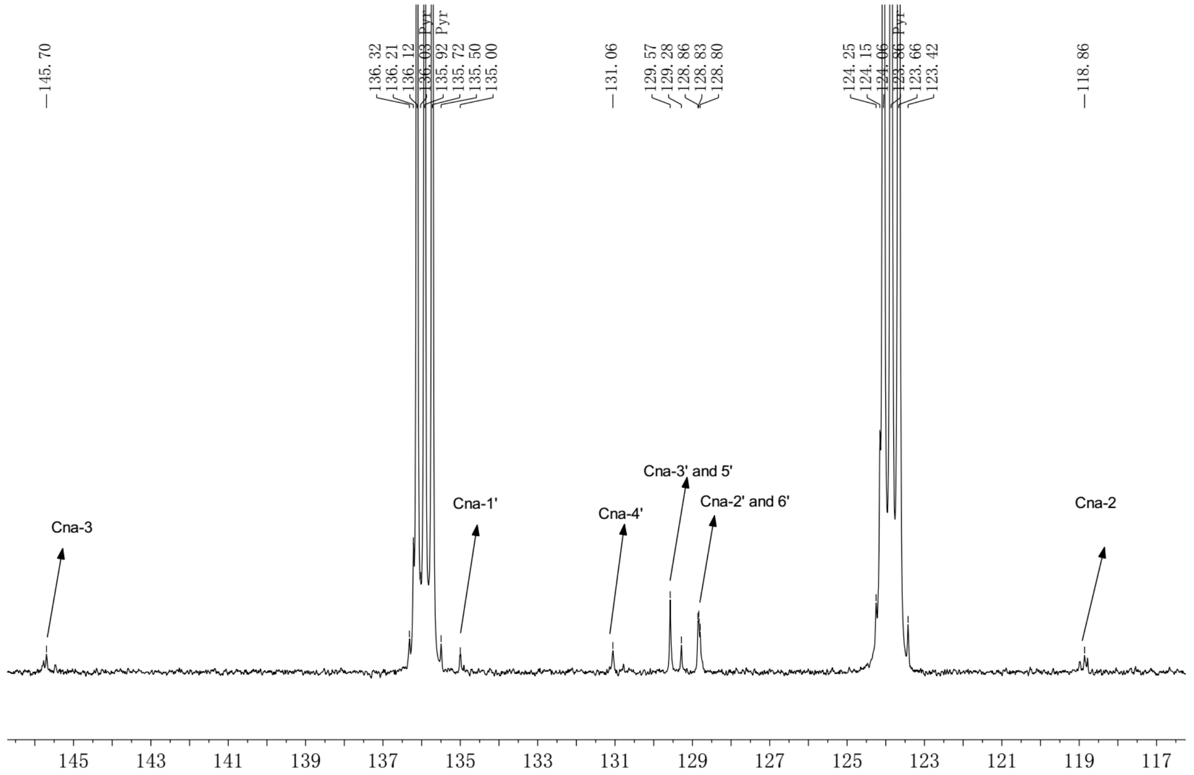

Figure S12-3. The ^{13}C-NMR spectrum of compound **3**

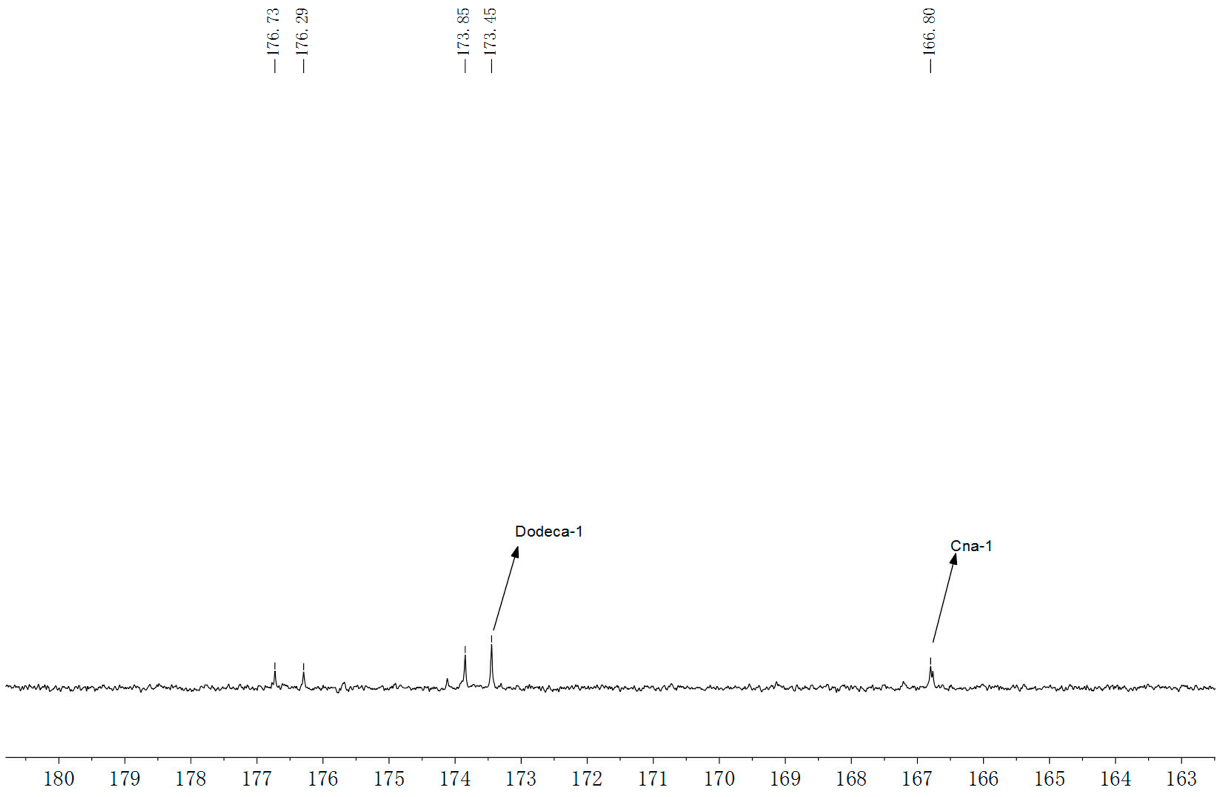

Figure S12-4. The ^{13}C-NMR spectrum of compound **3**

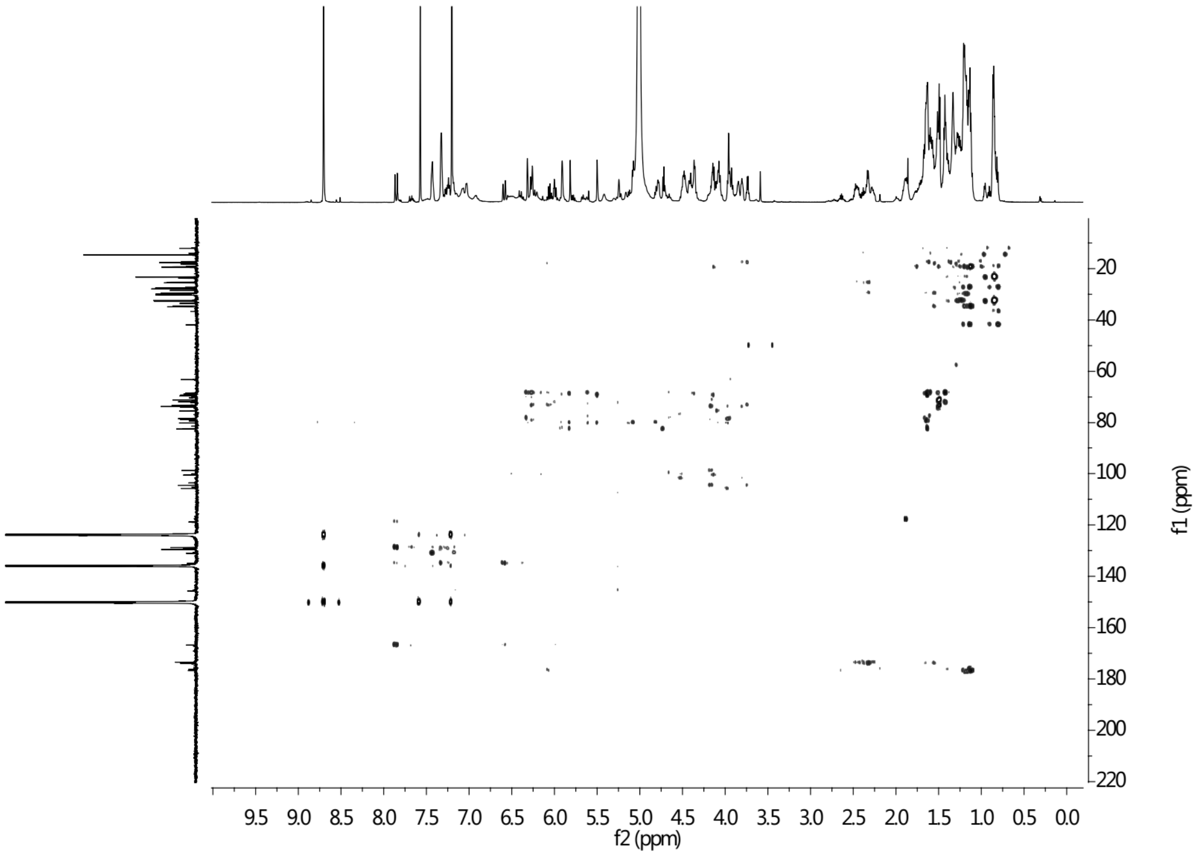

Figure S13. The HMBC spectrum of compound 3

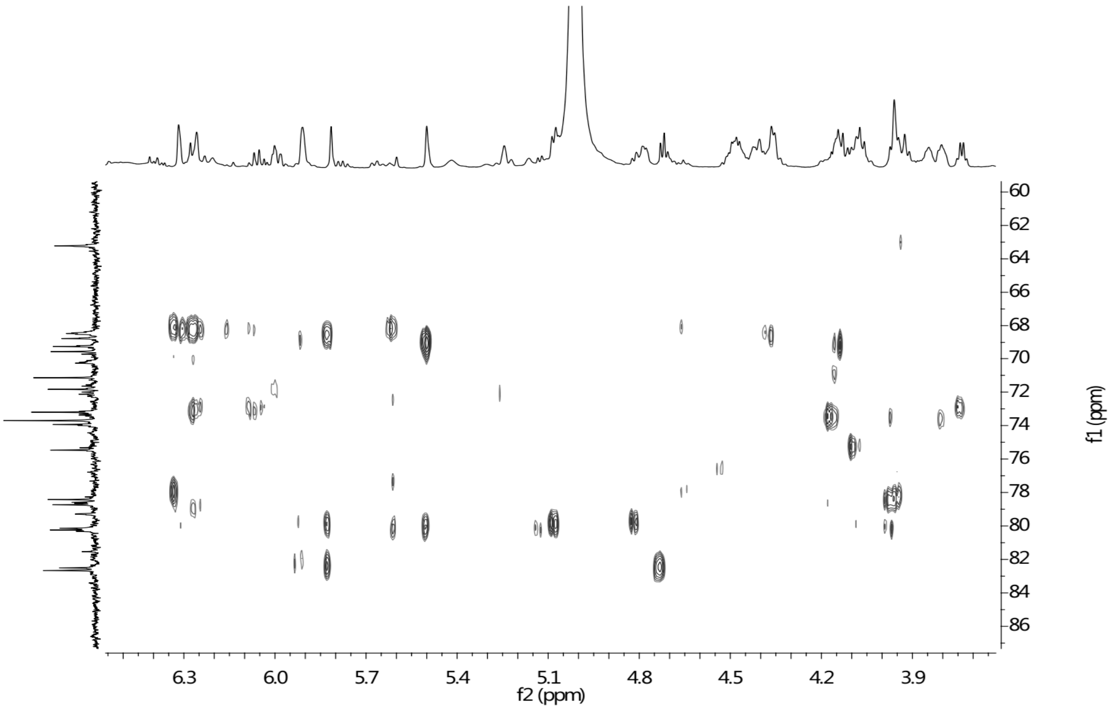

Figure S13-1. The HMBC spectrum of compound 3

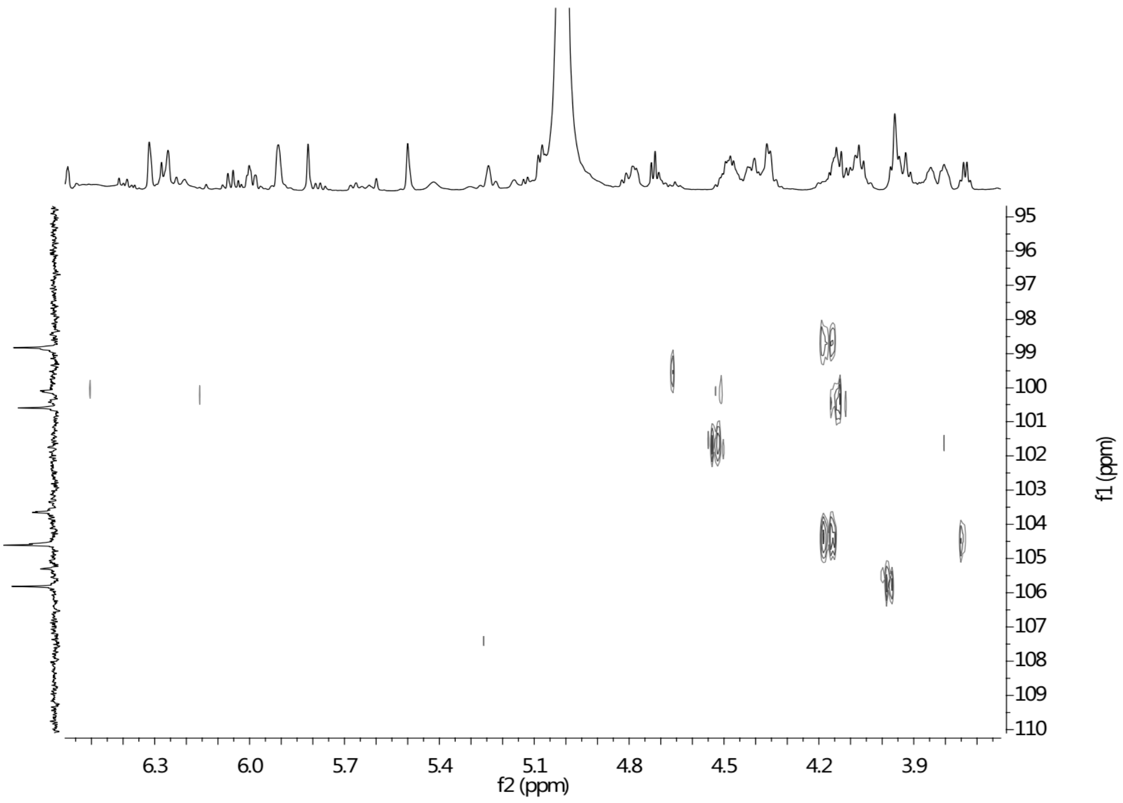

Figure S13-2. The HMBC spectrum of compound 3

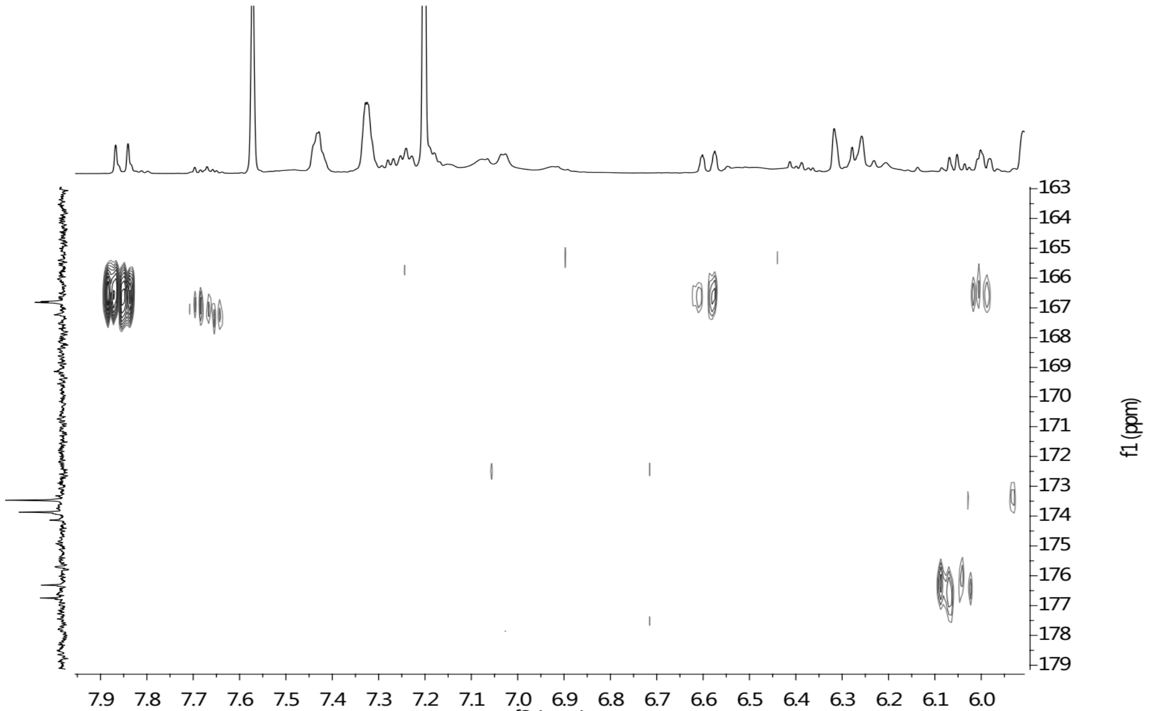

Figure S13-3. The HMBC spectrum of compound 3

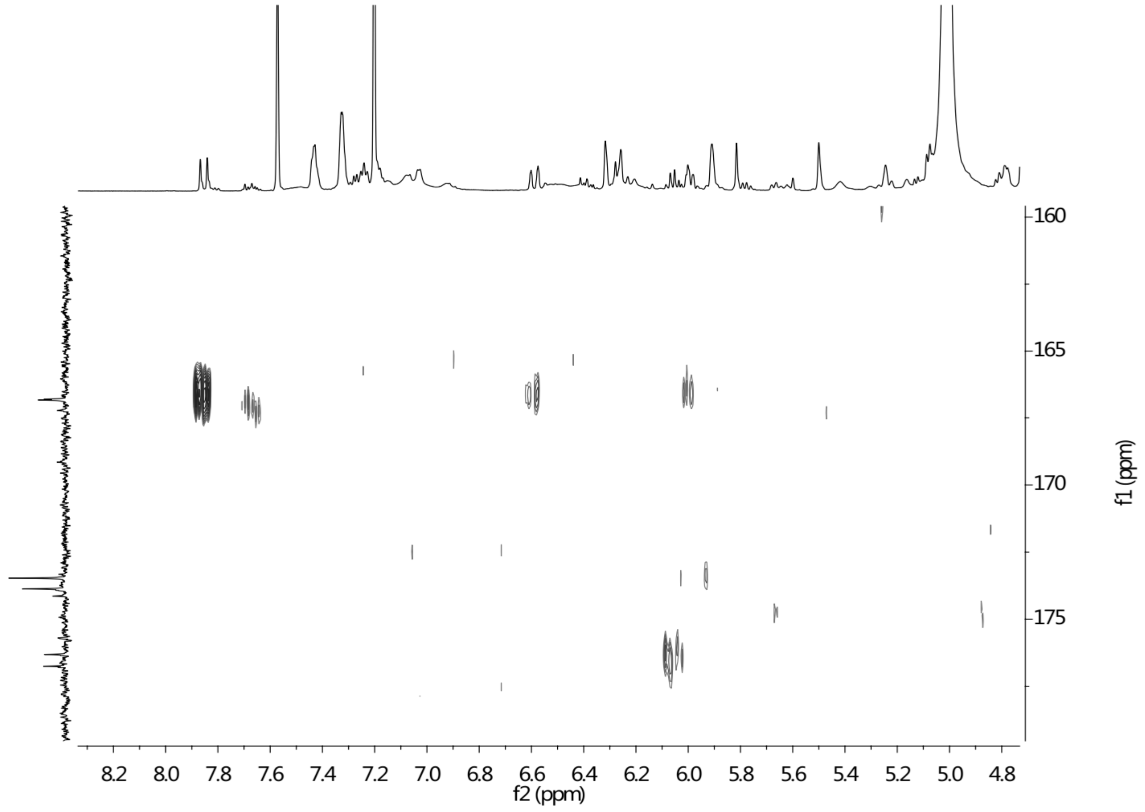

Figure S13-4. The HMBC spectrum of compound 3

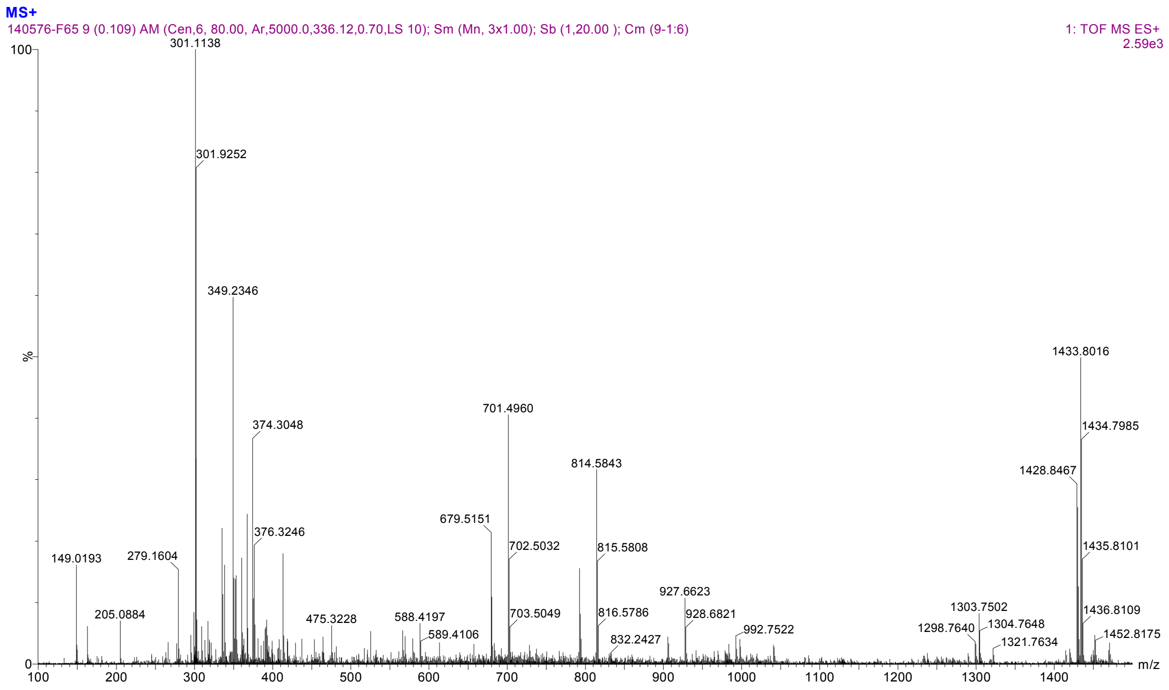

Figure S11. The HR-TOF-MS spectrum of compound **4**

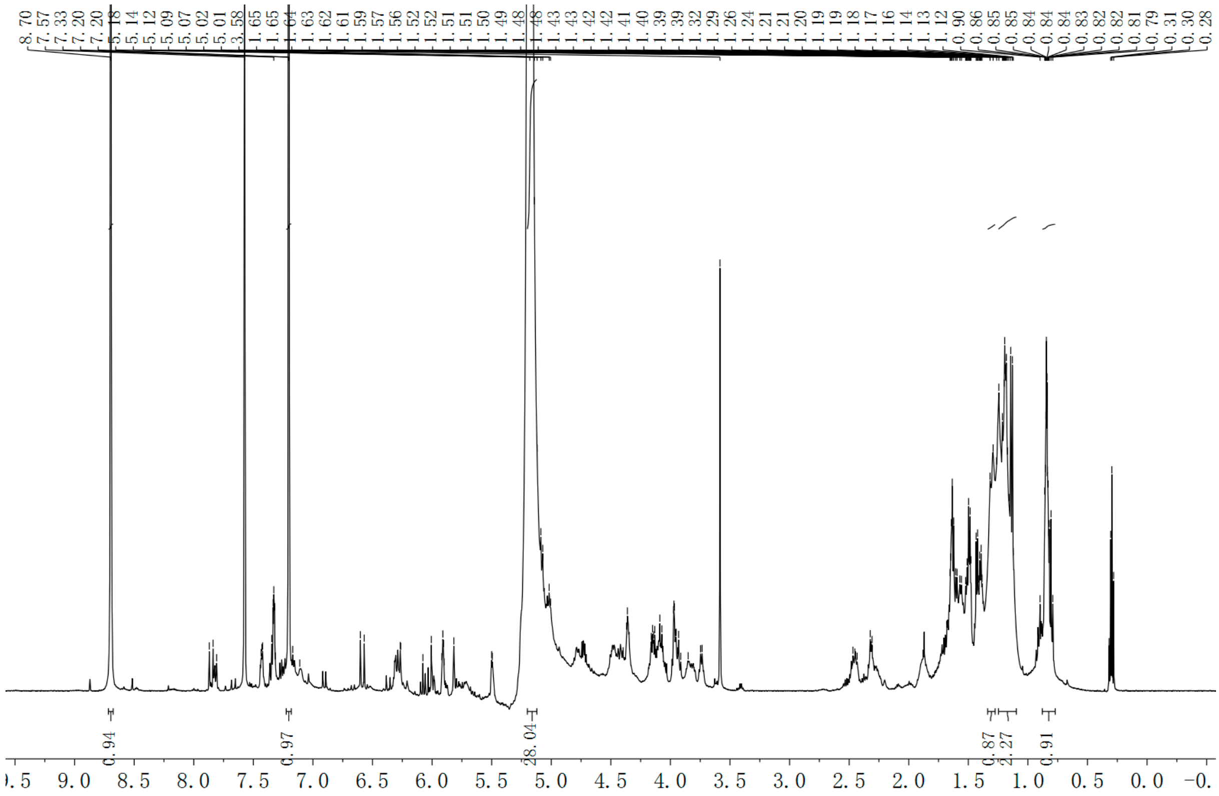

Figure S15. The ^{1}H-NMR spectrum of compound **4**

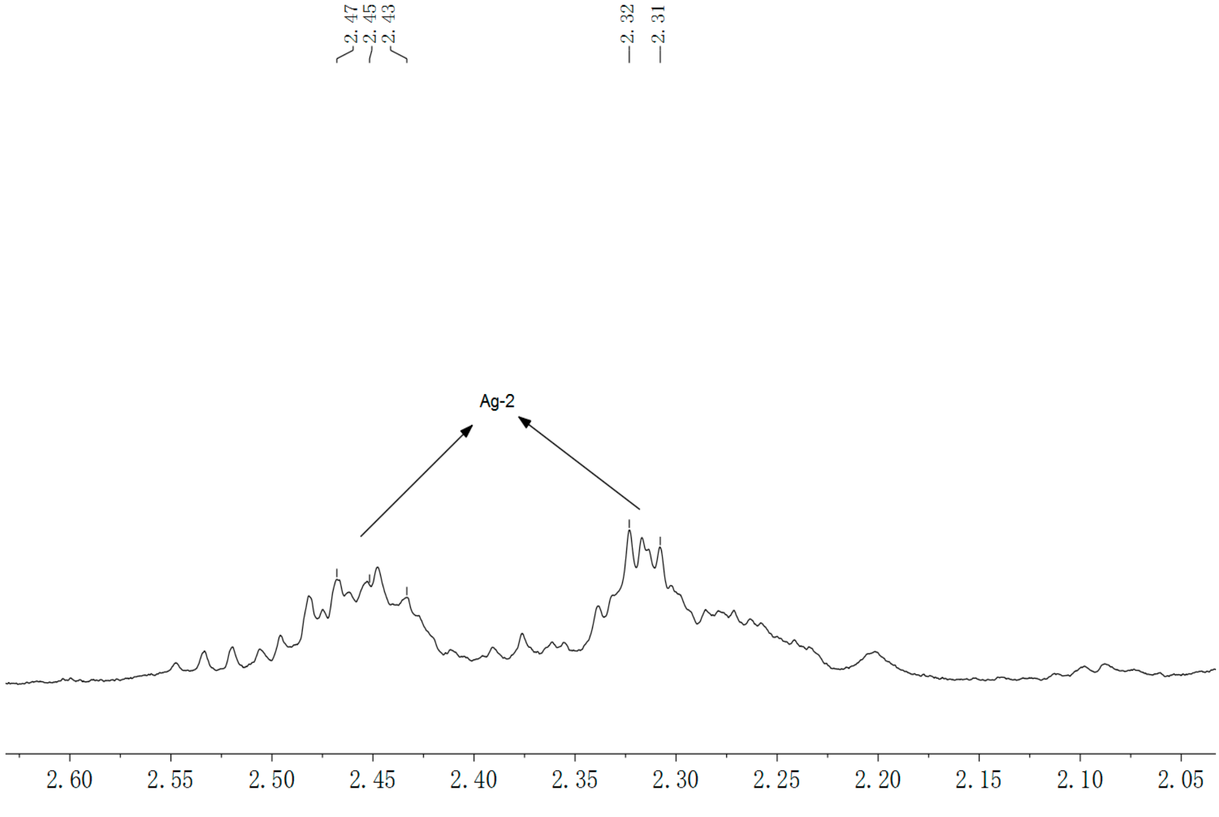

Figure S15-1. The ^{1}H-NMR spectrum of compound **4**

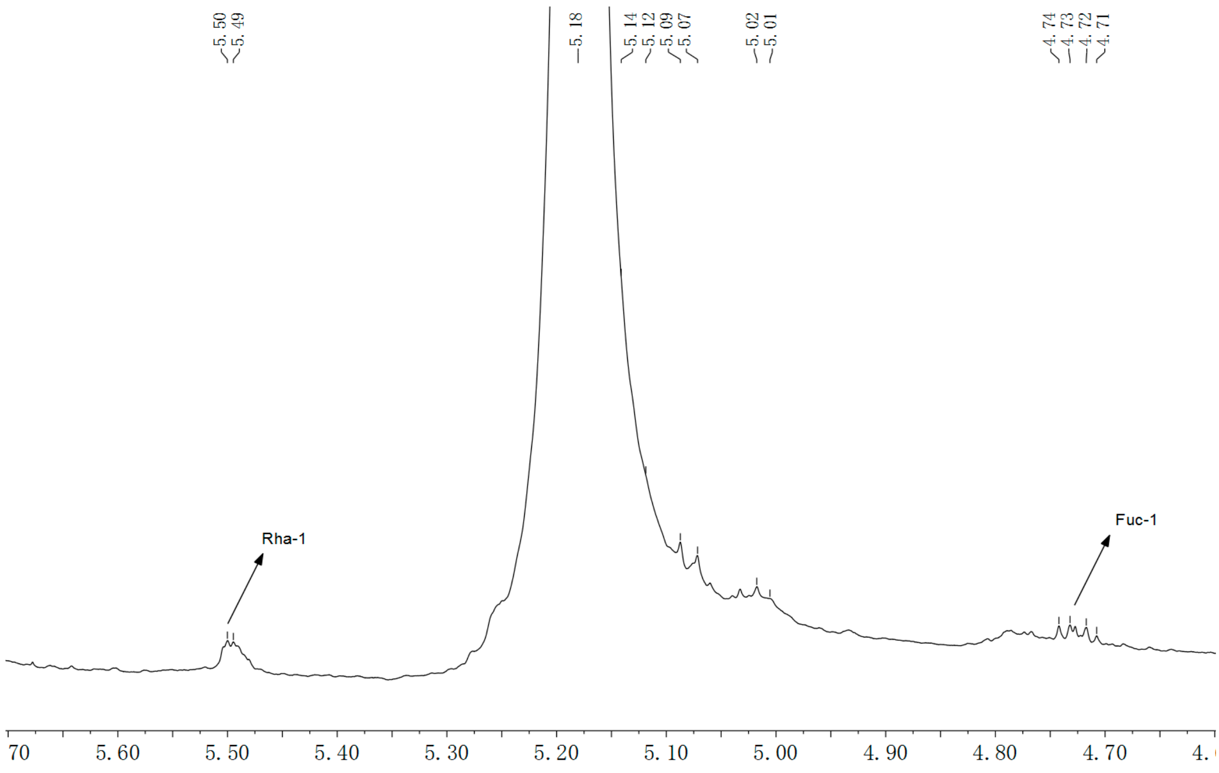

Figure S15-2. The ^{1}H-NMR spectrum of compound **4**

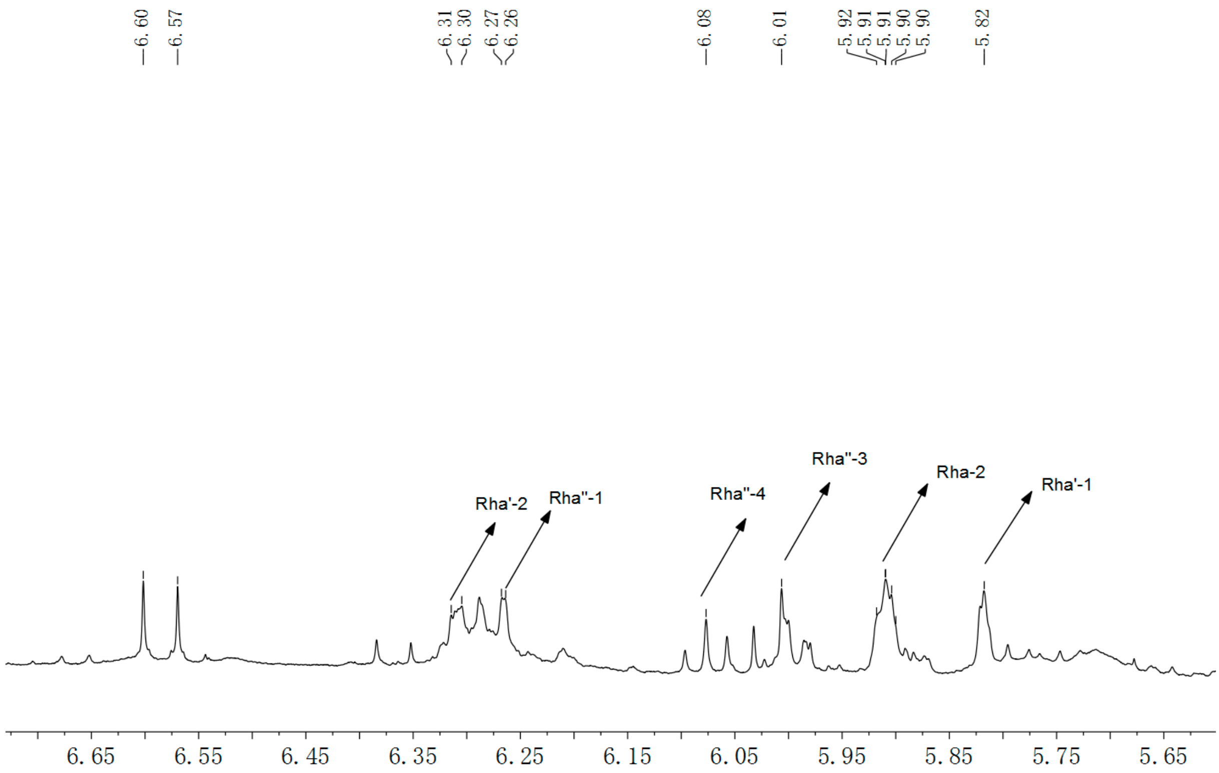

Figure S15-3. The ^{1}H-NMR spectrum of compound **4**

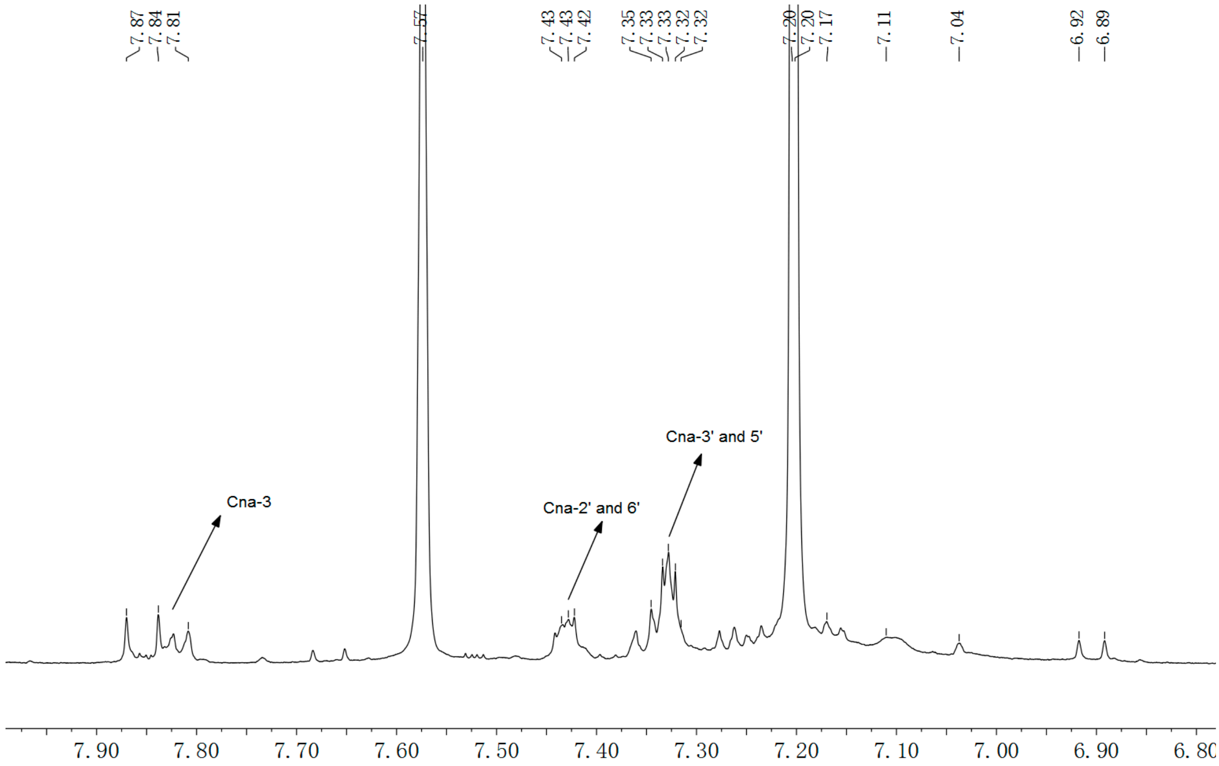

Figure S15-4. The ^{1}H-NMR spectrum of compound **4**

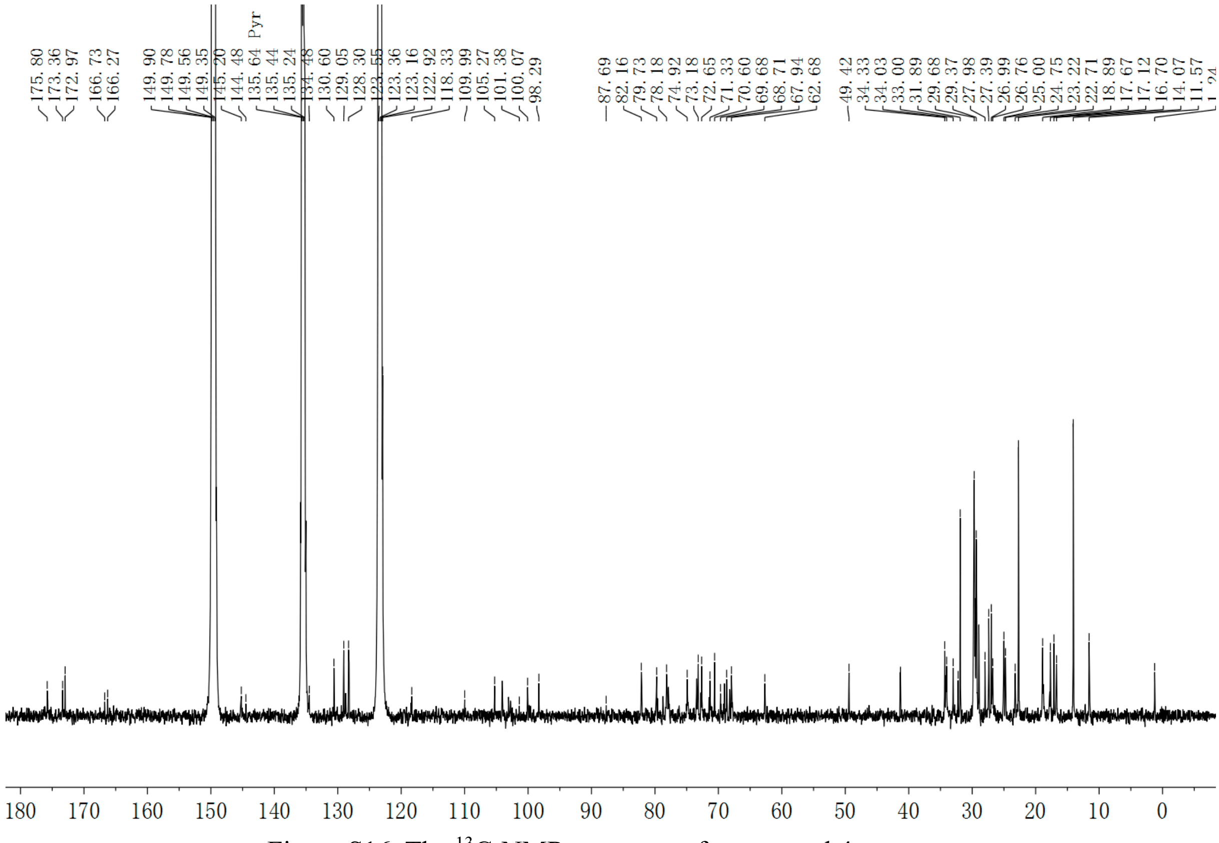

Figure S16. The ^{13}C-NMR spectrum of compound **4**

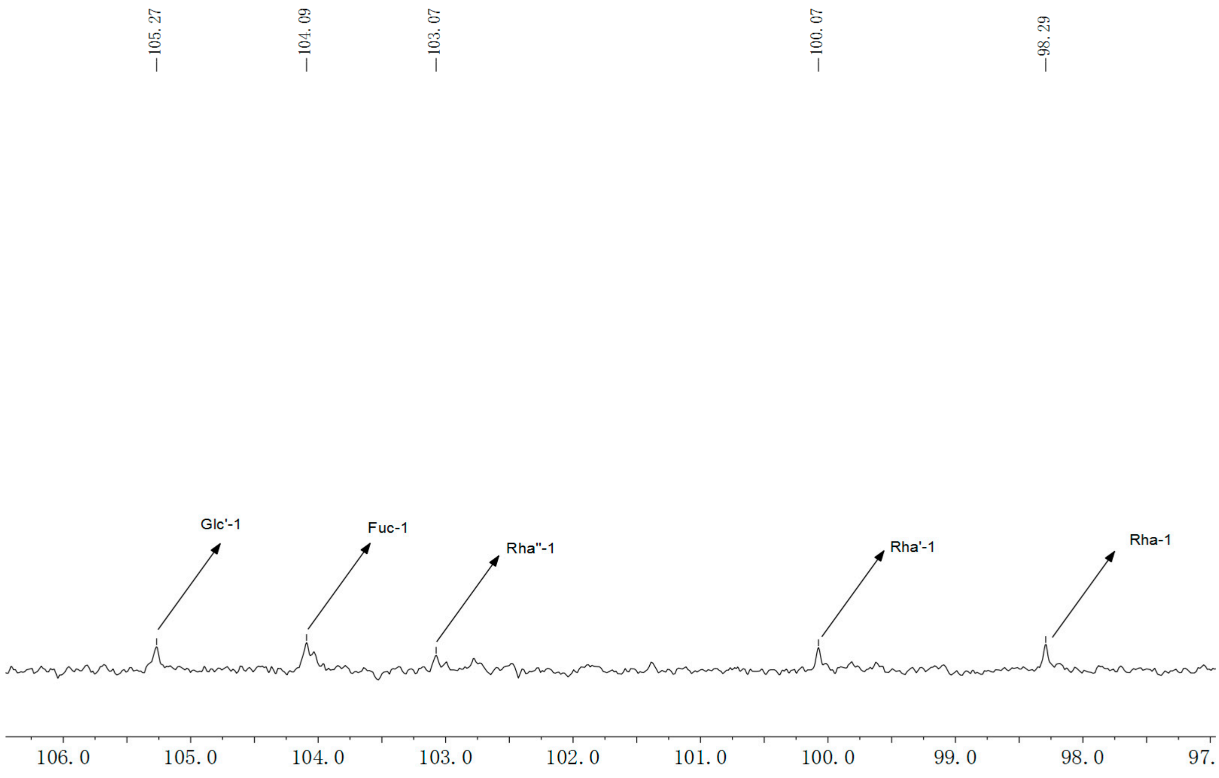

Figure S16-1. The ^{13}C-NMR spectrum of compound **4**

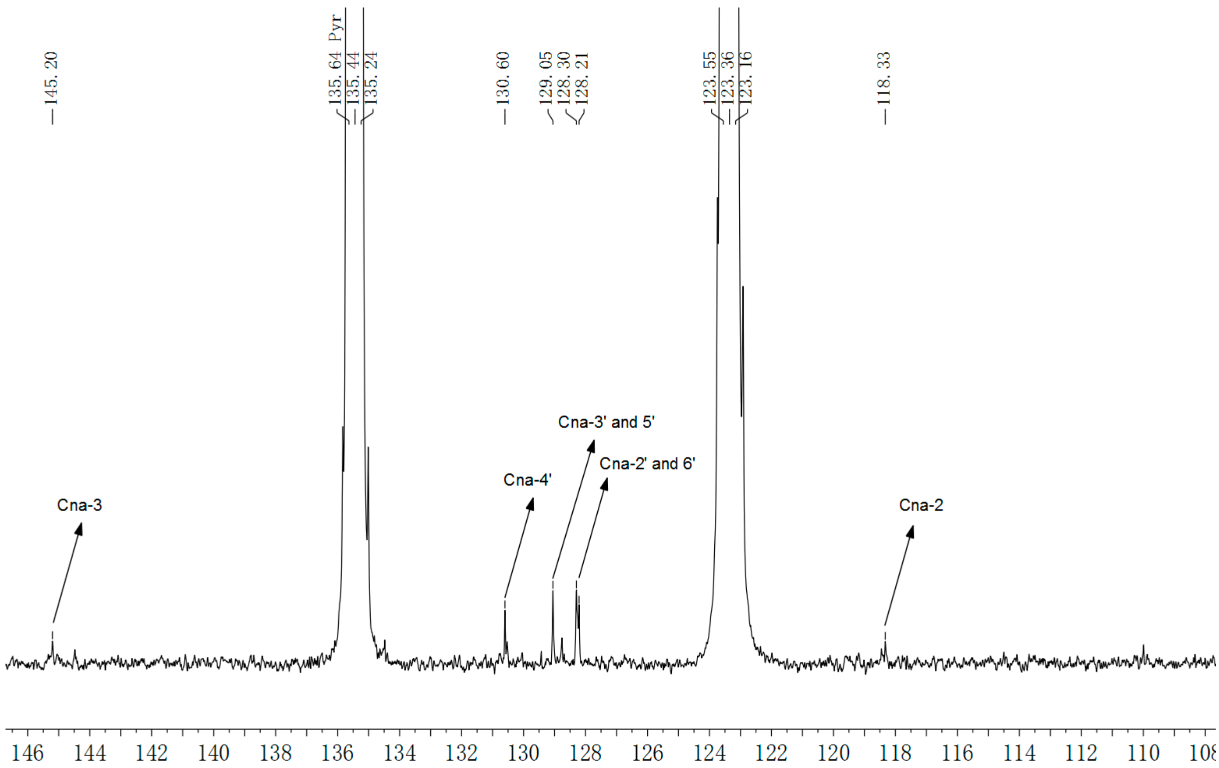

Figure S16-2. The ^{13}C-NMR spectrum of compound **4**

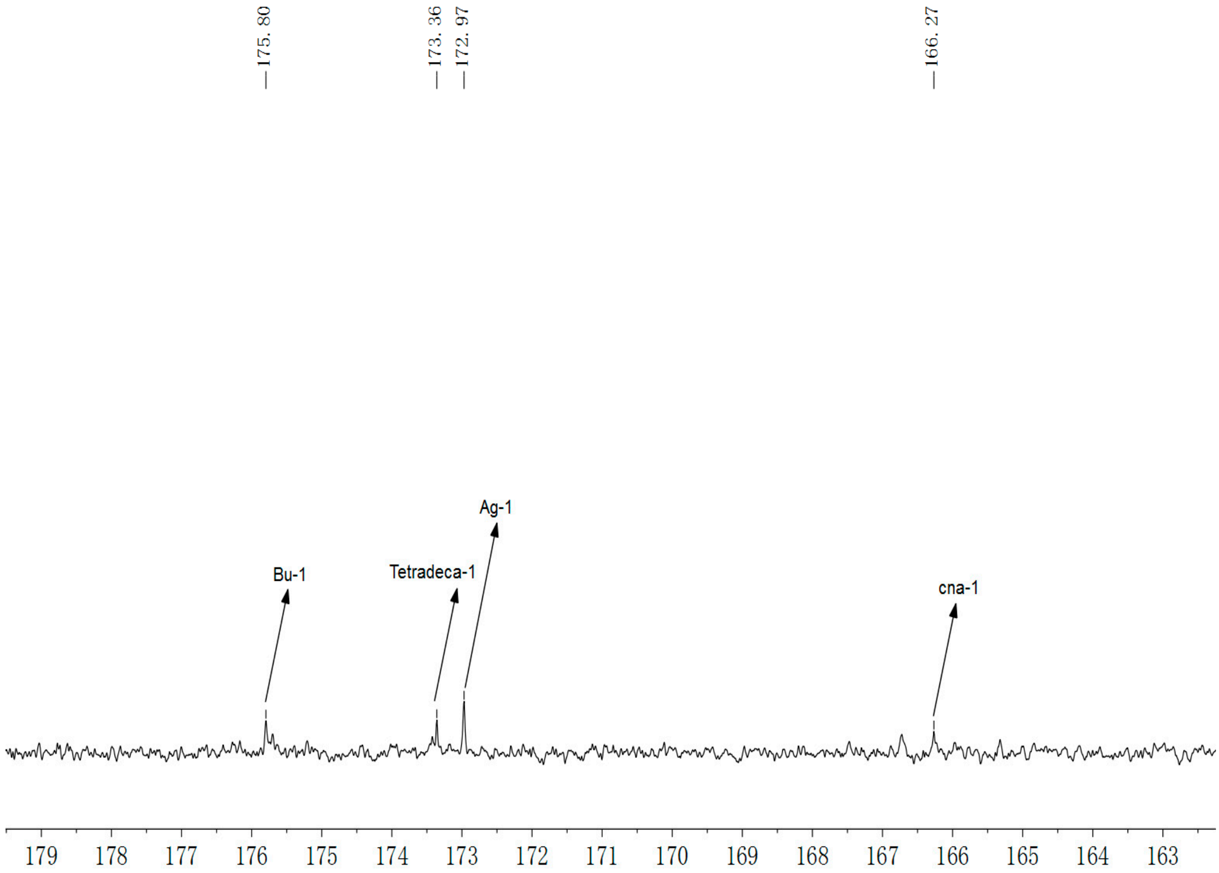

Figure S16-3. The ^{13}C-NMR spectrum of compound **4**

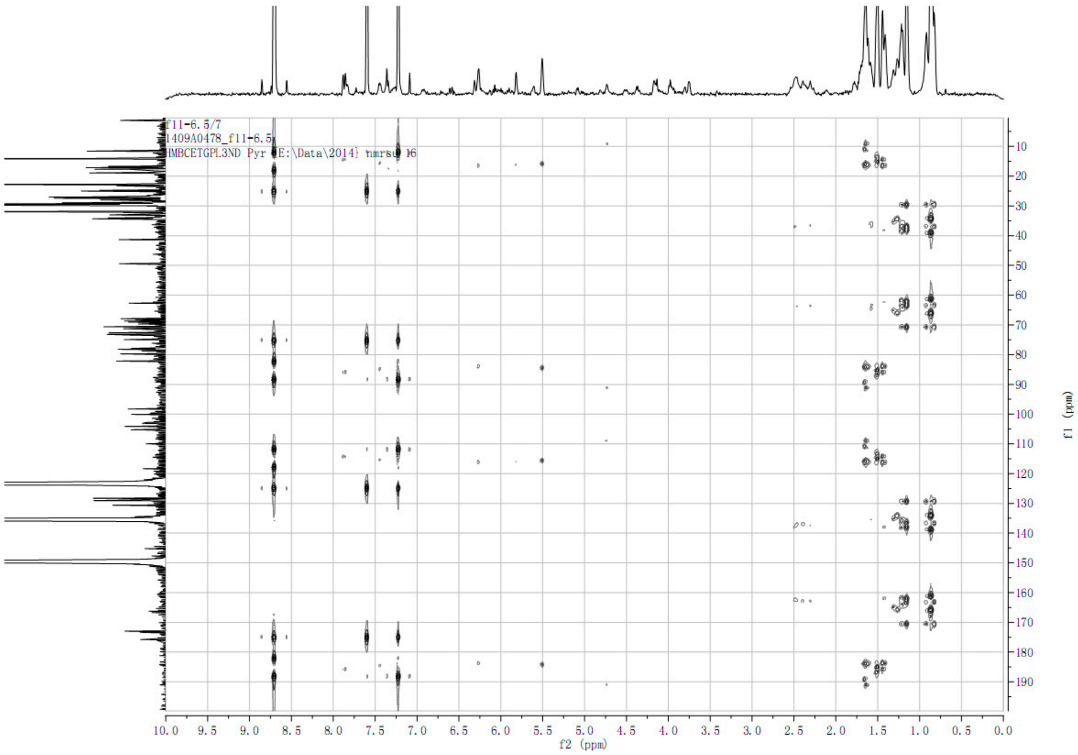

Figure S17. The HMBC spectrum of compound **4**
